# Digital Lifestyle Interventions to Support Healthy Gestational Weight Gain: Scoping Review

**DOI:** 10.2196/71548

**Published:** 2025-11-14

**Authors:** Renée A Otte, Lucie Duracher, Ozge Demir, Hanne A A Spelt

**Affiliations:** 1 Research and Advanced Development, Philips Mother & Child Care and Women's Health, High Tech Campus 37, Eindhoven, 5656AE, The Netherlands, 31 402730404; 2 Clinical Affairs Personal Health, Philips Mother & Child Care and Women's Health, Eindhoven, The Netherlands

**Keywords:** pregnancy, gestational weight gain, digital lifestyle interventions, behavioral change techniques, scoping review, mobile phone

## Abstract

**Background:**

Digital lifestyle interventions hold promise in supporting healthy gestational weight gain (GWG) during pregnancy. However, clarity on their key design and implementation features remains limited. The prevalence of excessive GWG and its associated maternal and infant health risks makes understanding the landscape of digital intervention characteristics critical.

**Objective:**

This scoping review aimed to map current literature on digital lifestyle interventions designed to promote healthy GWG and to identify intervention characteristics, including behavior change techniques (BCTs), used across these interventions, with particular attention to patterns in design and implementation features across studies reporting positive outcomes.

**Methods:**

We systematically searched PubMed, Embase, Cochrane, and Web of Science for peer-reviewed studies published between 2014 and 2024. Studies were included if they described interventions with at least 1 digital component targeting GWG. Studies on high-risk pregnancies, nonhuman participants, protocols without results, abstracts, gray literature, and non-English publications were excluded. Data extraction covered study characteristics, theoretical frameworks, timing, duration, frequency, delivery modes, and BCTs applied. The landscape of intervention characteristics was mapped, including descriptive analysis of features that appeared across different study outcomes.

**Results:**

A total of 44 studies met the inclusion criteria: 23 primary data articles (pilot studies, randomized controlled trials, etc) and 21 secondary data articles (meta-analyses, systematic reviews, etc). Primary studies showed that interventions were more likely to achieve intended outcomes when they started earlier, lasted longer, and combined digital and in-person components. Five BCTs were commonly present across interventions achieving positive outcomes: goal setting (outcome; 71%), discrepancy between current behavior and goal (43%), self-monitoring of behavior (86%), social support (unspecified; 71%), and credible source (71%). Secondary studies supported these findings, identifying several helpful features: starting before midpregnancy, long duration with high intensity, in-person contact, and BCTs related to goal setting, action planning, feedback on, and monitoring of behavior. However, primary studies showed gaps in reporting practices, with many details lacking about design and implementation features, such as BCTs. This converged with secondary studies reporting insufficient detail in the reviewed primary literature, limiting interpretation and replication potential.

**Conclusions:**

This scoping review maps digital interventions for GWG and identifies key patterns in intervention design and implementation. Evidence suggests that interventions may be more promising when combining digital delivery with in-person components and incorporating BCTs related to goal setting, self-monitoring, and social support. This review provides a comprehensive mapping of BCT usage and other intervention features, highlighting approaches associated with positive outcomes. However, significant gaps in reporting practices limit evidence synthesis. The findings can inform the design of digital interventions for managing GWG by identifying potentially successful design and implementation features. Future research should prioritize standardized reporting practices and evaluate interventions in underserved populations, including health care desert communities, to enhance the evidence base.

## Introduction

### Background

Pregnancy represents a critical window of opportunity for improving health outcomes for both mother and child. This transformative period catalyzes significant changes in a woman’s conception of self and social roles, fundamentally altering her capabilities, opportunities, and motivations for health behavior modification [1-3]. Throughout this paper, we use the term “woman’ to refer to individuals assigned female at birth, while acknowledging and respecting all gender identities. As a stage of transition, pregnancy presents an optimal time for encouraging healthy lifestyles, including weight management, with benefits that may persist well beyond pregnancy [4,5].

Current recognized medical guidelines, such as those by the Institute of Medicine (IOM), emphasize the importance of appropriate gestational weight gain (GWG) depending on prepregnancy BMI (ie, underweight BMI <18.5 kg/m²: 12.5‐18 kg; normal weight BMI 18.5‐24.9 kg/m²: 11.5‐16 kg; overweight BMI 25‐29.9 kg/m²: 7‐11.5 kg; and obese BMI ≥30 kg/m²: 5‐9 kg) for improved maternal and fetal outcomes [6,7]. Deviation from these recommendations – either excessive or insufficient GWG – has been associated with adverse outcomes. For women, these include increased risk of gestational diabetes, hypertensive disorders of pregnancy, cesarean delivery, and postpartum weight retention. For infants, risks encompass preterm birth, inappropriate birth weight, macrosomia, and increased likelihood of childhood obesity [8-10].

Despite the well-documented health implications, achieving recommended GWG remains a challenge for many women. Globally, 18%-30% of pregnant women gain insufficient weight, while 37%-51% exceed recommendations [8,11]. This challenge stems from multiple factors: many women are unaware that healthy weight gain targets differ per individual and depend on their preconception BMI [12,13], and even if women are acquainted with recommendations, they may not know how to operationalize them [14]. For example, the majority of pregnant women fail to meet dietary recommendations, such as those for vegetables, fats, and grains [13,15,16], and fall short of achieving the recommended 150 minutes of moderate-intensity aerobic activity per week [17-19]. In addition, common pregnancy symptoms such as nausea, fatigue, and pain, along with lack of knowledge and limited access to information, form additional barriers to maintaining healthy lifestyle practices [14,20-22].

Therefore, pregnant women require support in following healthy lifestyles and achieving healthy GWG. While health care professionals are ideally positioned to deliver this support through prenatal visits [3], they often face significant implementation barriers despite their motivation to address these guidelines [23,24]. For example, they may lack time, access to quality resources, training, and organizational support and perceive their efforts as being ineffective [23-25]. Consequently, many pregnant women report receiving either insufficient or inaccurate counseling on these topics, while they may desire it [6,14,26].

This issue is further exacerbated in maternity care deserts, where many women receive minimal or no prenatal care [27-29]. For example, between 2017 and 2023, in several low- and middle-income countries, less than 60% of pregnant women received at least 4 antenatal care visits while the World Health Organization recommends a minimum of 8 [3,30]. The problem extends to high-income countries, for example, the United States, where over 2.3 million women live in areas without obstetric services, with an additional 3 million having limited access to maternity care [27].

### Digital Lifestyle Interventions for Managing Healthy GWG

Digital lifestyle interventions have emerged as a promising solution to the challenges of supporting healthy GWG. Several studies have shown the potential of using digital lifestyle programs in a pregnant population. For example, Redman et al [31] reported that their eHealth intervention combining digital and nondigital components positively impacted healthy GWG, while Feng et al [32] found reduced GWG in participants using a smartphone-based intervention compared with matched controls. However, some interventions have not achieved their intended outcomes [33,34], while others have shown differential effects depending on prepregnancy BMI classifications [35,36]. Despite these mixed outcomes, digital lifestyle interventions offer unique advantages in transcending geographical and social barriers, offering cost-effective, scalable support on top of usual care [28,37-39].

Literature reveals considerable variability in how digital lifestyle interventions are designed and implemented, which contributes to the inconsistent results observed across studies. Whether digital behavior change interventions achieve their intended outcomes depends on multiple factors working in concert, necessitating mapping of intervention characteristics, theoretical foundations, and reported findings to better understand the current landscape of approaches. Even among interventions that have shown promise, uncertainty persists regarding which specific design components and implementation features may contribute to their success.

Some studies suggest there are no clear optimal specifications for implementation features of GWG management interventions, such as duration, contact frequency, intensity of use, delivery format (group vs individual), or dietary approach [40]. Conversely, other research indicates that factors like the timing of intervention initiation, delivery modes (digital-only vs digital-mixed), and the type and frequency of digital components may influence outcomes [37,41]. A possible explanation for these mixed findings is how behavior change interventions are described: variation in reporting practices impedes our understanding of intervention mechanisms, evidence synthesis, and the development of more effective interventions [42].

### Design and Implementation of Digital Lifestyle Interventions

Understanding the specific components that contribute to intervention success requires examination of both design and implementation features. Digital lifestyle interventions are a form of behavior change interventions, defined as programs that aim to change behavior with a clear objective and target group [43]. When designing interventions to change behavior, researchers draw on established theories to understand which components are most likely to produce change and what contextual factors might strengthen or weaken the intervention’s impact. The design of these interventions incorporates multiple behavior change techniques (BCTs), which represent the smallest active ingredients of an intervention capable of inducing behavior change [43-45]. BCTs are typically implemented based on theory, and understanding their proposed mechanisms of action may illuminate the processes by which BCTs influence behavior [46]. For example, the BCT “goal setting (behavior)” – defined as setting or agreeing on a goal defined in terms of the behavior achieved, such as agreeing on a 3-mile daily walking goal – works through increasing behavioral self-regulation.

To come to standardized reporting, researchers created a standardized classification system called the Behavior Change Technique Taxonomy to systematically identify and classify the techniques used to change behavior and better understand what makes behavior change interventions effective. Systematically identifying and quantifying BCTs used across interventions can help characterize intervention intensity to some degree, identify common patterns or clusters, and validate theory-based concepts [44,45]. This approach has proven value in previous reviews of interventions targeting smoking cessation [47] and physical activity (PA) [48].

The extent to which a BCT can achieve its intended purpose may vary depending on the target population, the specific behavior being addressed, and how the intervention is implemented [43]. As previously noted, implementation features such as timing, duration, frequency, and delivery modalities are critical as they represent the practical aspects of how interventions are delivered in real-world settings. It is therefore important to further explore how these features interact with BCTs to influence outcomes.

For healthy GWG interventions, implementation features are particularly important given the time-sensitive nature of pregnancy and the need to accommodate evolving physiological and psychological states. Research indicates that key implementation features influencing outcome include the timing of initiation (preconception, early pregnancy, or later stages), intervention duration (spanning the entire pregnancy or focusing on specific trimesters), frequency of delivery (daily contact vs weekly check-ins), and delivery modes (digital-only platforms vs hybrid approaches combining digital and face-to-face elements). The choice of a digital platform itself introduces additional implementation considerations (eg, user interface design, accessibility across devices, integration with existing health care systems, and the level of personalization offered) which are beyond the scope of this paper.

The relationship between these design and implementation features and positive outcomes for healthy GWG remains unclear without a systematic evaluation of existing evidence. Understanding which BCTs are most used, how they are typically implemented, and whether certain implementation approaches are associated with better outcomes requires a comprehensive mapping of the current intervention landscape.

### Goal of This Study

To address the knowledge gap surrounding the characteristics and implementation of digital lifestyle interventions for healthy GWG, we conducted a scoping review focusing on interventions developed between 2014 and 2024. Our objectives were to systematically map intervention characteristics and identify design and implementation features that appear to be associated with positive outcomes. We examined theoretical foundations, intervention timing, duration, frequency, delivery modes, and the BCTs used across studies.

We addressed 2 primary research questions. First, what is the scope and nature of evidence on digital lifestyle interventions for healthy GWG? Second, what design and implementation features characterize digital interventions that report positive outcomes?

By addressing these questions, we aim to synthesize existing knowledge about intervention components and provide insights that may inform future research and development of digital interventions for supporting healthy GWG in addition to usual care. This paper presents a descriptive and narrative analysis of our findings.

## Methods

### Overview

We used a scoping review methodology to map the breadth of existing evidence and identify patterns in the design and implementation of digital lifestyle interventions. This methodology is particularly well-suited for exploring the extent of existing literature, mapping and summarizing evidence, identifying key characteristics, and informing future research directions [49]. This approach was most appropriate given the heterogeneity in digital intervention designs and reporting practices in this field, where the goal was to map evidence patterns rather than to conduct a quantitative synthesis for clinical decision-making.

Our review was guided by Chapter 10 of the Manual for Evidence Synthesis from the Joanna Briggs Institute, specifically the section on scoping reviews [49]. For drafting this paper, we followed the PRISMA-ScR (Preferred Reporting Items for Systematic Reviews and Meta-Analyses extension guidelines for scoping reviews) [50] (Checklist 1). A review protocol was developed for internal use but was not registered.

### Eligibility Criteria

To define eligibility criteria, we used the Population, Concept, and Context framework [49], with population being “pregnant women” and concept “digital lifestyle interventions for managing gestational weight gain.” With “digital” interventions, we refer to those with at least 1 digital component (eg, app, SMS text message, and website), potentially in addition to nondigital components (eg, paper booklets and in-person sessions). The context was left unspecified to allow for the inclusion of studies conducted across diverse health care settings and populations.

Both articles on primary data (eg, pilot studies and randomized controlled trials [RCTs]) and on secondary data (eg, reviews and meta-analyses) were eligible. To ensure relevance to current digital health practices, the search was limited to articles published within the last 10 years. A preliminary scan of literature before 2014 indicated that earlier studies either did not fit our inclusion criteria or lacked sufficient focus on digital components. Given the rapid evolution of digital health technologies, we considered this time frame appropriate for capturing the most relevant and applicable evidence. Articles were excluded from our selection if they were not available in full in English; were not based on human participant data (eg, computer-generated data); were study protocols without results; were not related to pregnancy; did not include GWG as a primary or secondary outcome; were not peer-reviewed publications; did not investigate digital lifestyle interventions; or focused on high-risk pregnancies, such as placenta previa and gestational diabetes (to maintain focus on general GWG management rather than condition-specific interventions). A complete list of inclusion and exclusion criteria is provided in Multimedia Appendix 1.

### Search Strategy

The search strategy was developed collaboratively by all 4 authors. To retrieve a comprehensive set of relevant studies, we translated our main research question into 3 broad search terms: “smartphone application,” “mHealth,” and “gestational weight gain.” These terms were used to search 4 electronic databases: PubMed, Embase, Cochrane, and Web of Science. These databases were selected for their extensive coverage of biomedical and life science literature. Our search strategy confirmed the appropriateness of this selection: while each additional database yielded some unique records, few of these met our inclusion criteria, suggesting that our chosen approach achieved near-saturation of the relevant literature.

The search strings were adapted to meet the syntax requirements of each database. Refer to Multimedia Appendix 2 for the exact strings used per database. The searches in PubMed, Embase, and Cochrane were carried out on March 8, 2024, and the search in Web of Science was completed on March 25, 2024. To match eligibility criteria, the searches were limited to articles published in English between 2014 and 2024 and within the defined population and context scope. Search results from each database were exported as .csv files and merged into a single Microsoft Excel file for screening and deduplication.

### Article Selection

First, duplicates were removed using a custom Python script. It recoded each article’s title to an ID by removing all punctuation marks, turning all capitals into lowercase, and removing all spaces. These IDs were used to identify and delete duplicate entries. The remaining, nonduplicate articles were imported and stored in a Zotero library (Corporation for Digital Scholarship) for further screening.

Then, each article was screened independently by different pairs of the 4 authors using the eligibility checklist, examining titles, abstracts, and results sections. Ineligible articles were assigned exclusion criterion codes to ensure traceability. Any disagreements between reviewers were resolved through structured discussion, with a third author consulted when consensus could not be reached.

To enhance comprehensiveness, we also searched the internet for follow-up publications of excluded study protocols and hand-searched the reference lists of all included articles. Any additional articles identified through these methods underwent the same rigorous screening and selection process.

### Data Extraction

Selected articles were read in full by at least 2 of the 4 authors in various combinations, and relevant information was extracted. If an article was deemed ineligible at this stage, it was assigned an exclusion criterion code and removed from the database. All exclusion decisions were discussed and agreed upon by at least 2 authors to ensure consistency and transparency.

Extracted data items included study type, study goal, population details, sample size, theoretical foundation, timing, duration, frequency, delivery mode, BCTs used, and results. The full list of data items and their definitions, along with the methodology for handling assumptions and simplifications for certain data items to ensure consistency across studies, is provided in Multimedia Appendix 3.

### Data Synthesis

Data synthesis was conducted through a multistage descriptive and narrative analysis led by RAO, with support from the other authors. First, for primary and secondary data articles, we analyzed extracted study characteristics (publication year, country, study design, and so on) to map the scope and distribution of the evidence base. These findings were visualized using frequency tables.

Second, for primary data articles, we analyzed intervention characteristics including theoretical foundations, timing of initiation, duration, frequency, delivery mode, and BCTs. Third, for nonpilot primary data articles and secondary analysis of RCT data and meta-analyses, we categorized reported study outcomes as successful in achieving intended outcomes (yes or no) based on the study authors’ conclusions. We compared intervention characteristics across these outcome categories to identify patterns and potential distinguishing features. Throughout this data synthesis process, we used constant comparison methods to identify patterns, contradictions, and gaps in the evidence base.

## Results

### Overview

**Figure 1.**
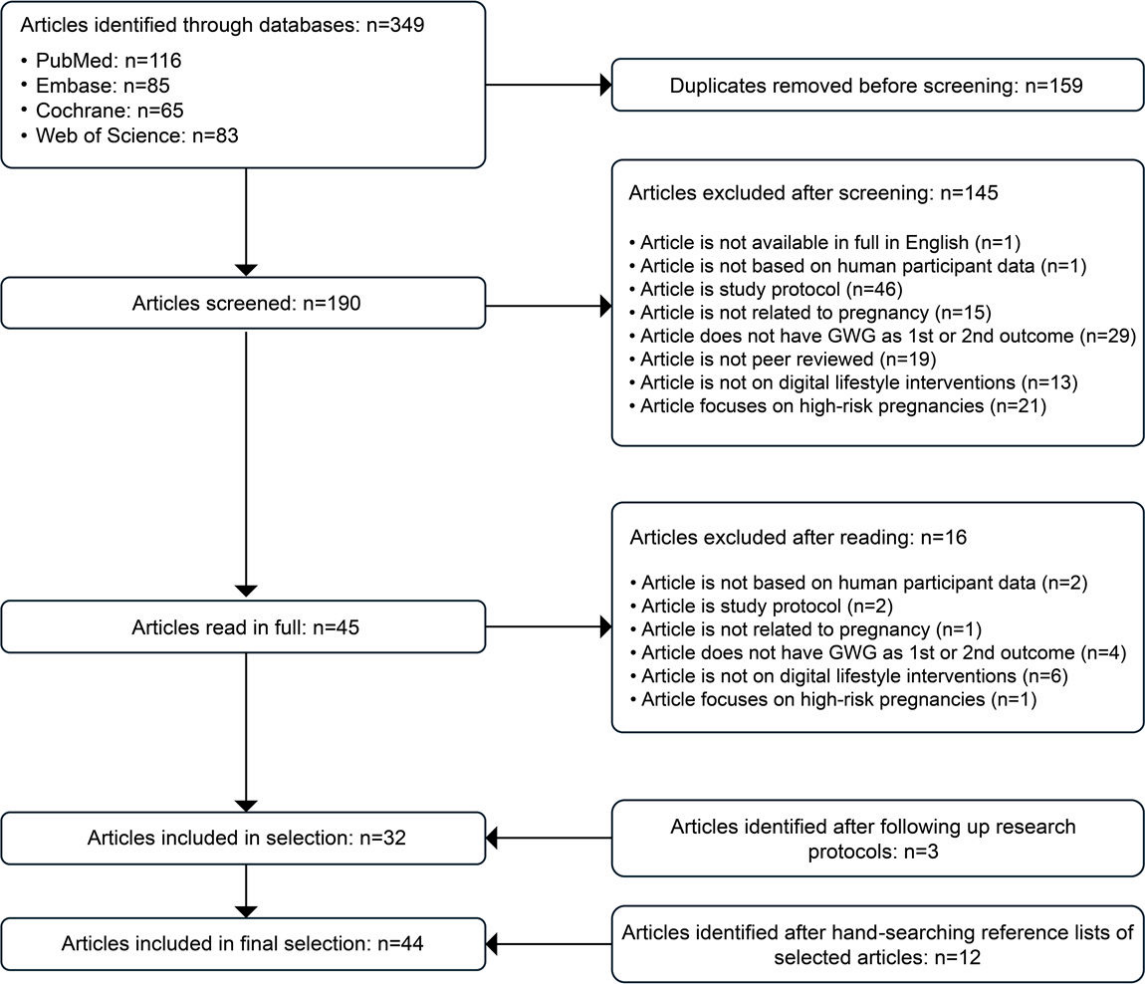
Flow diagram of identification and selection of articles. GWG: gestational weight gain.

We identified 349 potentially eligible articles through database searches, with an additional 15 articles found through research protocols (n=3) and hand-searching reference lists (n=12). Following full-text screening, 44 articles met the eligibility criteria: 23 primary data articles and 21 secondary data articles (meta-analyses, systematic reviews, and scoping reviews that included articles on digital lifestyle interventions). Figure 1 shows the article identification and selection flow. The complete data extraction file can be found in Multimedia Appendix 4.

### Descriptives of Primary and Secondary Data Articles

#### Primary Data Articles

**Figure 2.**
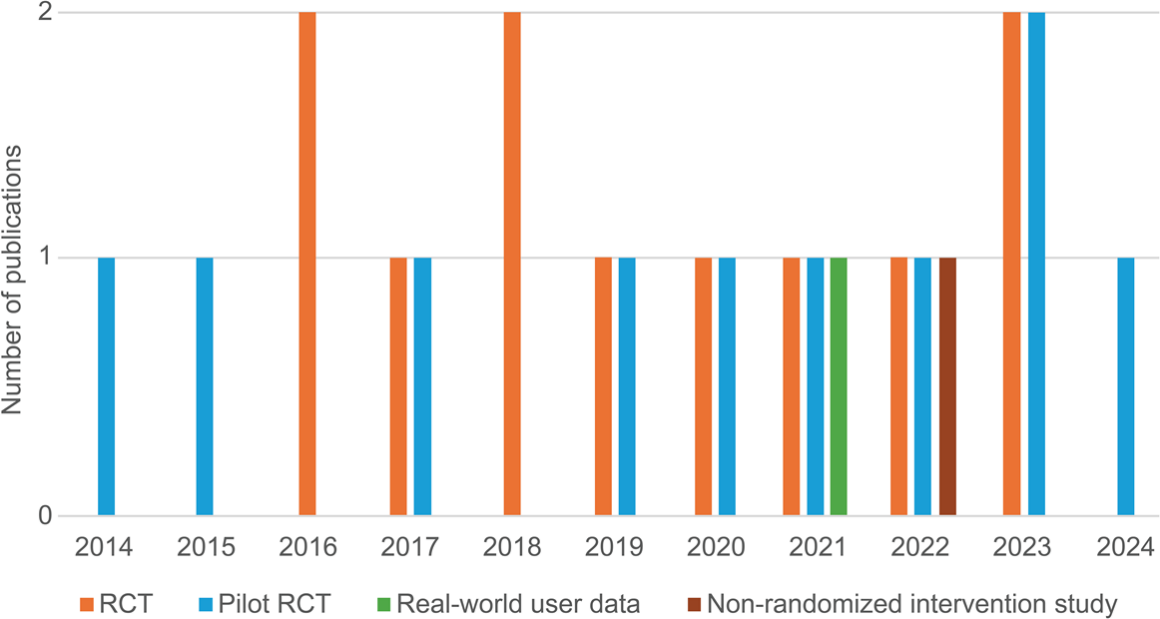
Distribution of the different types of the 23 primary data studies per year since 2014. RCT: randomized controlled trial.

The 23 primary data articles included 10 pilot RCTs (43%) [33,51-59], 11 full RCTs (48%) [9,31,32,34,35,60-65], 1 real-world user data study (4%) [66], and 1 nonrandomized intervention study (4%) [67]. These studies reported on data gathered between 2011 and 2022, with 6 studies (26%) collecting data (partially) during the COVID-19 pandemic [32,35,59,64,65,67]. As illustrated in Figure 2, pilot RCTs were conducted in most years except 2016 and 2018. From 2016 onwards, full RCTs were also conducted yearly, although these were not follow-ups of the pilot studies. A notable peak in RCTs occurred between 2016 and 2019, potentially reflecting increased interest and funding in digital health interventions during that period.

Studies were conducted in 10 different countries: United States (n=13, 57%) [9,31,34,51,56-63,66], Australia (n=2, 9%) [53,55], Canada (n=1, 4%) [67], China (n=1, 4%) [32], Finland (n=1, 4%) [33], Singapore (n=1, 4%) [54], Spain (n=1, 4%) [64], Sweden (n=1, 4%) [35], Taiwan (n=1, 4%) [65], and the United Kingdom (n=1, 4%) [52]. Sample sizes ranged from 12 to 15,468 participants (median 68, average 916). One study using commercial app data from over 15,000 women drove the high mean [66]. Table 1 summarizes the 23 primary data articles, with complete details in Multimedia Appendix 4.

**Table 1.** Summary of the included primary data articles.

Study	Study type	Sample size	Intervention	Timing^a^ (in gestational age)	Delivery medium	Results^b^
Digital-mixed interventions						
Soltani et al [52]	Pilot RCT^c^	14	IG^d^ and CG^e^. IG received the MOMTech intervention, including 2 daily SMS text messages, 4 appointments with a healthy lifestyle midwife, goal setting for diet and PA^f^, and use of self-monitoring diaries.	14‐16	SMS text message, face-to-face	IG had lower mean GWG^g^ than CG (mean 5.65, SD 4.6 kg vs mean 9.74, SD 7.2 kg; not tested statistically). Fewer women in IG exceeded IOM^h^ guidelines (4, 28% vs 6, 50%; not tested statistically).
Smith et al [61]	RCT	51	IG and CG. All participants had access to a website on which CG could view general recommendations on diet and PA. IG had additional access to PA goal-setting modules, problem-solving tools, journal, calendar, and community forum.	10‐14	Website, pen-and-paper	Compared with CG, IG significantly increased sustained PA (+54 min on average; *P*<.05). IG had higher mean GWG than CG (mean 13.6, SD 5.6 kg vs mean 11.2, SD 5.1 kg, Cohen *d*=0.45). The amount of activity performed by women in IG was not sufficient to prevent eGWG^i^.
Redman et al [31]	RCT	54	CG, IG remote, and IG in-person. IG app included personalized dietary intake prescription, weight self-monitoring, activity tracking with a pedometer, receipt of health information, and continuous personalized feedback from counselors.	10‐13	App, face-to-face	Both IGs together had lower overall GWG than CG (mean 9.2, SD 0.9 kg vs mean 12.8, SD 1.5 kg; *P*=.04). In-person IG gained less overall weight compared with CG (mean 8.0, SD 1.3 kg vs mean 12.8, SD 1.5 kg; *P*=.04). Remote IG gained less overall weight compared with CG, but this was only a trend (mean 10, SD 1.3 kg vs mean 12.8, SD 1.5 kg; *P*=.07). Rate of GWG was lower in in-person IG compared with CG (0.31 kg/wk vs 0.49 kg/wk; *P*=.04), and comparable with remote IG. Proportion of women with excess GWG was significantly lower in both IGs compared with CG (10/18, 56%, *P*=.03 and 11/19, 58%, *P*=.04 vs 11/13, 86.4%, respectively; OR^j^ 0.25, 95% CI 0.04-1.45).
Willcox et al [53]	Pilot RCT	91	IG and CG. Both received CAU^k^, including brochures with advice on diet and PA. IG also received tailored SMS text messages, access to a website, video messages, chat room interaction, and guidance from trained researcher who educated them on nutrition, PA, and GWG goals, and helped them track weight and set goals.	13‐17	SMS text messages, social media, pen-and-paper, face-to-face	There was a significant difference in GWG between groups, with IG participants gaining an average 7.8 (SD 4.7) kg and CG average 9.7 (SD 3.9) kg (*P*=.04).
Van Horn et al [62]	RCT	280	IG and CG. CG received biweekly newsletters and links to publicly available maternity websites. IG received DASH^l^ diet and PA coaching. A commercially available app was used for self-monitoring of diet and PA, with additional support provided through telephone, SMS text message, and email.	15	App, face-to-face, mHealth^m^ tools	IG gained significantly less weight than CG (mean 10, SD 6 kg vs mean 12, SD 6 kg, *P*=.02; Cohen *d*=0.33). Fewer women in IG exceeded IOM guidelines (96/140, 67% vs 119/141, 84%, *P*=.004).
Altazan et al [63]^n^	RCT	54	CG, IG remote, and IG in-person. IG app included personalized dietary intake prescription, weight self-monitoring, activity tracking with a pedometer, receipt of health information, and continuous personalized feedback from counselors.	10-13	App, face-to-face	Proportion of women exceeding GWG guidelines was 56.3% (18/32) in IG and 81.8% (9/11) in CG (*P*=.17). Women in IG had less overall GWG as compared with CG (mean 8.7, SD 0.9 kg vs mean 12.8, SD 1.5 kg; *P*=.03; Cohen *d*=3.33).
Darvall et al [55]	Pilot RCT	27	CG, app IG, and app+coach IG. All participants wore pedometers. In CG, the pedometer display was obscured and could not be synced. Participants in both IGs had a pedometer synced to their personal smartphone. They also received a 4-session behavioral change program.	13‐19	App, face-to-face, telephone, mHealth tools	There was no significant difference between groups in GWG: app IG: mean −5.46, SD 2.8 kg, *P*=.07; app-coach IG: mean −0.40, SD 2.8 kg, *P*=.89, both compared with CG.
Ferrara et al [9]	RCT	394	IG and CG. CG received CAU. IG received a program to improve GWG, diet, PA, and stress management, including in-person and telephone sessions.	8‐15	Pen-and-paper, face-to-face, telephone	IG had significantly lower GWG than CG (mean 10.21, SD 5.6 kg vs mean 12.36, SD 5.3 kg, *P*≤.001; Cohen *d*=0.39). Women in IG had significantly lower rates of GWG per week than women in CG (mean between-group difference −0.07 kg per wk, 95% CI −0.09 to −0.04, *P*<.001). Proportion of women exceeding guidelines for weekly GWG rate and total GWG was significantly lower in IG than in CG (96/199, 48% vs 134/195, 69%, RR^o^ 0.70, 95% CI 0.59-0.83, *P*<.001 and 80/199, 41% vs 128/195, 66%, RR 0.62, 95% CI 0.51-0.76, *P*<.001, respectively). Proportion of women meeting IOM guidelines for weekly GWG rate and total GWG was significantly higher in IG than in CG (65/199, 33% vs 46/195, 24%, RR 1.38*,* 95% CI 1.00-1.90, *P*=.049 and 69/199, 36% vs 42/195, 22%, RR 1.66, 95% CI 1.21-2.30, *P*=.002, respectively). Proportion of women gaining below IOM guidelines for weekly rate of GWG and total GWG was significantly higher in IG than CG (38/199, 19% vs 15/195, 8%, RR 2.49, 93% CI 1.44-4.31, *P*<.001 and 45/199, 23% vs 24/195, 12%, RR 1.84, 95% CI 1.17-2.87, *P*=.008, respectively).
Downs et al [56]	Pilot RCT	24	IG and CG. All participants received CAU and monitored their GWG, PA, and dietary intake. Participants wore 1 activity monitor daily and 1 in 2-week cycles to track PA. Intake was recorded via app on 2 weekdays and 1 weekend day. In addition, the IG received weekly dietitian meetings, individualized caloric goals, and educational booklets on diet and PA. Intervention dosage was reviewed and adjusted every 3‐4 weeks as needed.	8‐12	App, email, pen-and-paper, face-to-face, mHealth tools	IG gained 1.9 kg less than CG, but this was not significant (*P*=.43, 95% CI −6.6 to 2.9). In IG PA from pre- to posttest increased, while in CG it decreased. This difference was not significant (*P*=.48). Energy intake increased less from pre- to posttest for IG compared with CG (*P*=.02).
Thomas et al [57]	Pilot RCT	68	IG and CG. All participants received CAU. IG also set PA goals working up to 150 active minutes/week, wore accelerometer, and weighed themselves daily. They received monthly calls to review and reset goals, access to a website with collected data and GWG resources, and personal messages.	10‐12	SMS, email, website, pen-and-paper, telephone, mHealth tools	Participants in IG and CG had the same total GWG (+1.14 kg, 95% CI −0.71 to 3.00). Participants in IG and CG had the same rate of GWG (+0.03 kg, 95% CI −0.02 to 0.09).
Digital-only interventions						
Pollak et al [51]	Pilot RCT	33	Txt4Baby IG and PregCHAT IG. Txt4Baby IG received general pregnancy-related SMS text message 3 days/week. PregChat IG received personalized feedback through SMS text messages 3 days/week based on intake of sweetened beverages, fruits and vegetables, fast food, daily step count, and weight.	16	SMS text messages	Participants in IG gained 6 pounds less than those in CG, but this was not statistically significant (*P*=.24, 95% CI −15.9 to 4.0).
Herring et al [60]	RCT	66	IG and CG. All participants received CAU. IG additionally received guidance through personalized health coach calls, texts, and feedback; pedometer; and DVD; on energy intake, PA, and weight. Also, participants received education and shared updates in a Facebook (Meta) group.	8‐17	SMS text message, social media, telephone	IG was significantly less likely to exceed IOM guidelines than CG (10/27, 37% vs 19/29, 66%; *P*=.03). IG gained less weight in pregnancy than CG (mean 8.7, SD 6.6 kg vs mean 12.3, SD 6.4 kg; *P*=.046; Cohen *d*=0.55).
Olson et al [34]	RCT	1689	Placebo, pregnancy IG and postpartum CG, and pregnancy and postpartum IG. All participants received CAU and behavioral change tools on a website and app platform, but the placebo group did not receive weight gain tracker, diet and PA goal setting, and self-monitoring tool.	12‐20	App, email, website, mHealth tools	Authors reported no significant difference in proportion of women with excessive GWG in IG versus CG (542/1126, 48% vs 260/563, 46%, RR 1.09, 95% CI 0.98-1.20, *P*=.12).
Li et al [54]	Pilot RCT	26	IG and CG. All participants received standard dietary guidance for pregnancy during recruitment. IG also received 8 weeks of real-time food coaching via app. They could upload images of meals, drinks, or desserts and receive feedback and guidance from professional dietitians.	18‐20	App	More participants met guidelines in IG than in CG (4-wk follow-up: 7/12, 58% vs 8/15, 53%; 8 wk follow-up: 8/12, 67% vs 5/14, 36%; not tested statistically). Although not significant, IG had less GWG than CG at both 4- and 8-week follow-up (−0.15 kg, 95% CI −1.51 to 1.21, *P*=.83 and −0.08 kg, 95% CI −1.80 to 1.63; *P*=.92, respectively).
Litman et al [66]	Real-world user data	15,468	BabyScripts IG. App is provided through HCP^p^ to track GWG. Participants could enter weight manually or via a connected scale. App gives targeted, gestational-age-specific educational materials.	<20	App	Highly engaged participants had increased adherence to IOM guidelines (762/2555, 29.8% vs 302/3209, 9.4%, *P*<.001). A larger proportion of highly engaged participants adhered to IOM guidelines for rate of GWG in trims 2 and 3, compared with lowest engaged patients (325/2555, 12.7% vs 219/3209, 6.8%, *P*<.001).
Sandborg et al [35]	RCT	305	IG and CG. All participants received CAU. IG received a 6-month app program, encouraging healthy diet and PA. This included push notifications 4 times/week for information, support, strategies, guidance, encouraging information, and reminders.	13‐14	App, mHealth tools	No statistically significant effect on GWG between IG and CG (−0.2 kg, *P*=.62; Cohen *d*=0.28). No statistical difference in adherence to recommendations between IG and CG (67/134, 50% vs 68/137, 50%, *P*=.32). Results differed per BMI group: for women with BMI ≥25, GWG in IG was lower than for those in CG (−1.67 kg, *P*=.03). IG had better diet quality than CG (β-coefficient=0.27; *P*=.02).
Gonzalez-Plaza et al [64]	RCT	120	IG and CG. All participants received CAU. IG used a smartband-connected app to monitor PA and for communication with midwife, who provided personalized health guidance.	12‐28	App, SMS text messages, mHealth tools	Median GWG in IG was significantly lower than in CG (median 7.0, IQR 4-11 kg vs median 9.3, IQR 5.9-13.3 kg, *P*=.04; Cohen *d*=0.42). Adjusted mean GWG per week was significantly lower in IG than in CG (0.3 kg/wk vs 0.5 kg/wk, *P*=.008). IG had higher mean PA than the CG (1980 vs 1386 MET^q^ min/wk, respectively, *P*=.01).
Souza et al [67]	Nonrandomized intervention study	27	IG only divided into higher or lower app usage group that got access to SmartMoms Canada app, Google Fitbit, and Withings scale. App provided real-time feedback on nutrition, PA, sleep, and GWG. Participants were encouraged to use it daily.	12‐20	App, mHealth tools	Higher vs lower app usage group better adhered to GWG guidelines, but this was not statistically significant (Cramer V=0.21, *P*=.54). Higher vs lower app usage group had more moderate PA (mean difference 8.41, 95% CI 1.05-15.77, *P*<.05) and MVPA^r^ (mean difference 17.84, 95% CI 2.44-33.25, *P*<.05).
Chen et al [65]	RCT	80	IG and CG. All participants received CAU. IG used app and a wearable activity tracker. App features included prenatal history, goal setting, chart history, records, prenatal information, rewards, reminders, and prenatal tools.	17	App, SMS text messages, mHealth tools	Proportion of participants exceeding total GWG was not significantly different between CG and IG (8/37, 22% vs 14/43, 33%, *P*=.28). In trimester 2, significantly lower proportion of IG exceeded weekly GWG (18/37, 45% vs 29/43, 67%, *P*=.04). For trimester 1 and 3, they performed like CG. In trimester 3, obese women in IG had less total GWG and body weight than those in the CG (−8.8 kg, *P*=.04 and −5.4 kg, *P*=.02, respectively). This did not hold for trimesters 1 and 2.
Feng et al [32]	RCT	268	CG and IG. All participants received CAU. IG monitored weight, diet, and PA with an app. App contained sections for weight management (through weighing, diet, and PA), daily recordings (with feedback), data trends, reminders, and pregnancy education per gestational week.	6‐7	App	Overall median GWG in IG was significantly lower than that in CG (median 8.5, IQR 5.5-11 kg vs median 10.0, IQR 6-14 kg; *P*=.008; Cohen *d*=0.42).
Koivuniemi et al [33]	Pilot RCT	1038	Standard-app IG and enhanced-app IG. All participants recorded lifestyle habits (PA and diet), monitored changes by viewing graphs of their recordings, and received reminders for recording information via app. Enhanced-app IG received additional, nonpersonalized, motivating information on health-promoting lifestyle during pregnancy.	9‐20	App	Authors reported no significant differences in GWG or in changes in IDQ^s^ scores or MET scores. In IG, the proportion of women with regular eating frequency was lower in late as compared with early pregnancy (OR 0.47, 95% CI 0.22-0.98, *P*=.045). In CG, there was no such difference (OR 1.44, 95% CI 0.69-3.01, *P*=.33). Proportion of women with high and moderate activity decreased more in app nonusers than in frequent app users (OR 0.61, 95% CI 0.40-0.94, *P*=.03) and occasional app users (OR 0.55, 95% CI 0.32-0.97, *P*=.04).
Waring et al [58]	Pilot RCT	12	IG. Participants used a website to track diet, PA, and GWG, and an open community. Participants were encouraged to check their feed daily, post regularly themselves, track diet and PA, and weigh themselves weekly. Researchers posted messages daily and interacted asynchronously with participants.	14‐18	Website	The authors reported that 70% (7/10) of participants had eGWG, 10% (1/10) had inadequate GWG, and 20% (2/10) gained within the recommended ranges.
Mattson and Barger [59]	Pilot RCT	22	Historical CG, self-weighing IG (WA), and self-weighing+counseling IG (WC). WA and WC groups were asked to weigh themselves weekly. WC group also received 6×30 min online counseling on healthy diet.	10‐25	Telehealth system, mHealth tools	Participants from WC and WA combined gained less weight than those in CG, but this effect was not significant (−0.7 lb, *P*=.72). Participants in WC gained less than those in the WA, but this effect was not significant (−1.5 lb, *P*=.52). Participants from WC and WA combined who weighed themselves ≥6 times/wk gained less than those who weighed <6 times/wk, but this effect was not significant (−2.7 lb, *P*=.99)

a Timing of initiation of intervention.

b For nonpilot studies with a control group, effect sizes for differences in mean gestational weight gain are reported.

c RCT: randomized controlled trial.

d IG: intervention group.

e CG: control group.

f PA: physical activity.

g GWG: gestational weight gain.

h IOM: Institute of Medicine.

i eGWG: excessive gestational weight gain.

j OR: odds ratio.

k CAU: care as usual.

l DASH: dietitian-led dietary approaches to stop hypertension.

m mHealth: mobile health.

n This paper belongs to a series of papers reporting on the same study as the one by Redman et al [31]. Hence, it describes the same interventions.

o RR: relative risk.

p HCP: health care professional.

q MET: metabolic equivalent of task.

r MVPA: moderate-to-vigorous physical activity.

s IDQ: Index of Diet Quality.

#### Secondary Data Articles

**Figure 3.**
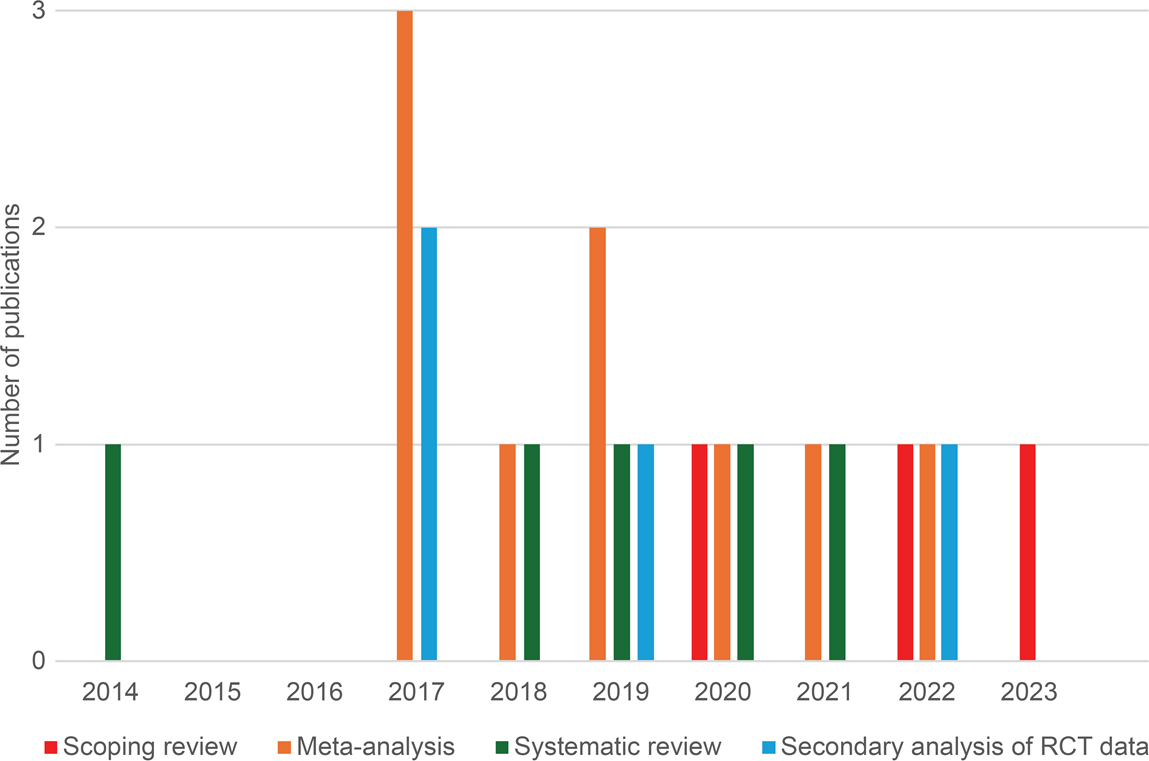
Distribution of the different research methodologies used in the 21 secondary data articles on (digital) interventions for gestational weight gain management since 2014.

The 21 secondary data articles included 9 meta-analyses (43%) [37,38,40,68-73], 5 systematic reviews (24%) [74-78], 3 scoping reviews (14%) [41,79,80], and 4 secondary analyses of RCT data (19%) [36,81-83]. Of the 9 meta-analyses, 4 (44%) investigated lifestyle interventions in general, while the other 5 (56%) specifically focused on digital lifestyle interventions. All systematic reviews targeted digital lifestyle interventions exclusively. Of the 3 scoping reviews, 2 (67%) addressed general lifestyle interventions and 1 (33%) examined digital interventions specifically [1]. Figure 3 illustrates the temporal trend in research methodologies used in the secondary data articles. Meta-analyses, the most prevalent approach, peaked in 2017 with 3 publications. Notably, 2 meta-analyses published in 2017 [38,69] specifically addressed the effectiveness of digital interventions for managing GWG, despite a limited number of RCTs on the topic at that time (Table 1). Systematic reviews maintained consistent usage throughout the search period. Scoping reviews were published less frequently overall but showed an increase after 2021, which roughly coincides with the publication of the PRISMA Extension for Scoping Reviews in 2018 [50]. Secondary analyses of RCT data were typically published 1-4 years following the original data collection.

The secondary data articles synthesized studies published between 1992 and 2022, incorporating 36 publications on average (range 7‐97, median 21), with an average sample size of 526 participants per article (range 53‐2833, median 89). Overlap with our primary data articles varied across the secondary data article types: meta-analyses shared an average of 3 articles (range 2‐6), systematic reviews 2.2 articles (range 0‐5), and scoping reviews 3 articles (range 1‐13) with our selection of 23 primary studies.

In terms of inclusion criteria, the majority of the secondary data articles primarily included RCTs or quasi-RCTs examining healthy lifestyles in pregnant women and GWG management, although systematic reviews allowed for wider inclusion criteria encompassing observational and qualitative studies.

The target populations were predominantly pregnant women in general, with exceptions including 3 articles (14%) focusing specifically on women with overweight or obesity [41,69,70], 1 article (5%) included both healthy women and those with (gestational) diabetes [71], and 1 article (5%) targeting users of pregnancy-related mobile phone interventions [78].

A summary of all included secondary data articles can be found in Table 2, with detailed data extraction available in Multimedia Appendix 4.

**Table 2.** Summary of the included secondary data articles.

Study	Study type	Target group	Overlap primary articles/number of articles included (%)	Results
O’Brien et al [74]	Systematic review	All healthy pregnant women (without pregnancy-related conditions)	0/7 (0)	Technology-supported lifestyle interventions in pregnancy hold potential as safe and sustainable adjunct to traditional health care models. Quality and quantity of published evidence to support use of such interventions is low. Findings raise the issue of uptake levels and sociocultural acceptance of such lifestyle interventions.
Graham et al [81]	Secondary analysis RCT^a^ data	Normal weight to moderately obese pregnant women	—^b^	CG^c^: 3 different types of patterns of app usage. IG^d^: 5 patterns. In CG, GWG^e^ outcomes did not differ by usage pattern. In IG, GWG outcomes did differ by usage pattern. In the lower income-normal BMI group, “almost consistent” or inconsistent trackers had a risk of eGWG,^f^ and “inconsistent” trackers gained more than “nonuser” usage pattern. In the higher income-normal BMI group, “consistent” trackers had lower risk of eGWG rate than “nonusers.” In the higher income-high BMI group, “consistent” trackers gained less than “nonusers.” Compared with participants with lower usage patterns, participants in higher usage patterns and higher income gained less, both in normal and high BMI subgroups (total mean GWG −1.83 kg, 95% CI −3.58 to −0.54). For participants with lower income, no such difference.
The International Weight Management in Pregnancy (i-WIP) Collaborative Group [72]	Meta-analysis	All pregnant women except those with GDM^g^	2/81 (2)	Based on IPD^h^ data, diet- and PA^i^-based IGs resulted in significantly less GWG compared with CG (mean GWG −0.70 kg, 95% CI −0.92 to −0.48). When supplementing IPD data with non-IPD data, the difference between IG and CG increased (mean GWG −1.1 kg, 95% CI −1.46 to −0.74), but so did heterogeneity. No evidence for differential intervention effects across subgroups.
Lau et al [69]	Meta-analysis	Overweight and obese pregnant women	2/17 (12)	Participants in IG had lower GWG than CG (GWG −0.63kg, 95% CI −1.07 to −0.20, *P*=.004). Electronic-based lifestyle interventions with in-person, phone, or combination of those formats were found effective for reducing GWG (*P*=.004 for all 3). No such effect for solely electronic-based platforms (*P*=.27). No significant effects for subgroup differences.
Olson et al [82]	Secondary analysis RCT data	Normal weight to moderately obese pregnant women	—	Of the total, 16.5% (58/351) of low-income women and 34.2% (187/547) of not–low-income women consistently tracked GWG. More highly educated, older, and White women were more likely to be consistent GWG trackers. Among not–low-income women, consistent GWG tracking was associated with 2.35 kg less GWG (95% CI –3.23 to –1.46, *P*<.001) and reduced risk of eGWG (RR^j^ 0.73, 95% CI 0.59-0.89, *P*=.002).
Sherifali et al [38]	Meta-analysis	All pregnant women	4/10 (40)	Meta-analysis on 6 studies showed nonsignificant reduction in GWG (mean GWG −1.62 kg, 95% CI –3.57 to 0.33, *P*=.10) after exposure to the intervention.
Overdijkink et al [76]	Systematic review	All pregnant women	3/29 (10)	mHealth^k^ lifestyle apps and mHealth medical apps seem feasible and acceptable. Evidence of effectiveness is limited because of small sample sizes. Formal guidelines for quality certification of apps need to be developed.
Walker et al [40]	Meta-analysis	All pregnant women except those with diabetes or GDM	4/89 (5)	Women in dietary IGs gained less than those in CG (mean GWG −3.27 kg, 95% CI –4.96 to –1.58, *P*<.001). Women in PA IGs gained less than those in CG (mean GWG −1.02 kg, 95% CI –1.56 to –0.49, *P*<.001). Women in lifestyle IG (diet and PA combined) gained less than those in CG (mean GWG −0.73 kg, 95% CI –1.17 to –0.29, *P*<.001). Women in eHealth IGs gained less than those in the CG (mean GWG −2.26 kg, 95% CI –3.84 to –0.69, *P*<.001). Interventions in groups with group components were effective more often (effective 62.5%, ineffective 37.35%; *P*=.02) than those delivered individually (effective 33.3%, ineffective 66.7%; *P*=.04). The study did not find optimal duration, frequency, intensity, delivery method, or diet for preventing eGWG.
Chan and Chen [73]	Meta-analysis	All pregnant women	3/16 (19)	Moderate effect in maternal weight control and maintaining optimal body composition by promoting lifestyle change and self-monitoring via mHealth apps and social media (Cohen *d*=0.45)
Mertens et al [77]	Systematic review	All healthy pregnant women	2/11 (18)	Technology-supported lifestyle interventions might affect GWG and PPWR^l^, but more research is needed for examining their effectiveness, usability, and critical features. Interventions positively influence GWG and PPWL,^m^ but results are not always significant. Furthermore, effects on PA and healthy eating are inconsistent.
Olson et al [36]	Secondary analysis RCT data	Normal weight to moderately obese pregnant women	—	Among women with normal BMI, setting ≥2 goals+engaging in self-monitoring was associated with less GWG (*P*=.03). Also, risk for eGWG reduced (*P*=.04). Among women with higher BMI, setting ≥2 goals was associated with greater GWG (*P*=.01), and with significantly increased risk for eGWG (*P*=.03).
Vincze et al [71]	Meta-analysis	Pregnant women with (gestational) diabetes	2/48 (4)	A total of 12 out of 25 studies (48%) reported significant reductions in GWG. Despite heterogeneity, pregnant women in IGs gained less weight than those in CGs (mean GWG −1.25 kg, 95% CI –2.10 to –0.40, *P*=.004).
Hussain et al [78]	Systematic review	All pregnant women who used pregnancy-related mobile phone interventions	5/28 (18)	In high-income countries, use of mobile phone–based health behavior interventions in pregnancy demonstrates correlation with positive beliefs, behaviors, and health outcomes. More effective interventions are multimodal in terms of features and tend to focus on healthy GWG.
Hutchesson et al [80]	Scoping review	All pregnant women	4/90 (4)	Majority of research on behavioral interventions for women of childbearing age focused on weight management during or after pregnancy. Research gap to support weight management in young adult females in preconception and unrelated to pregnancy to improve chronic disease health trajectories. Future research to examine delivery modes and mediums, optimal intervention duration and intensity, involvement of health care providers, and involvement of underrepresented populations should be considered for effectiveness and scalability.
Rhodes et al [68]	Meta-analysis	All pregnant women, except with issues that would preclude them from participating in diet- or PA-based intervention	5/11 (50)	No significant benefit of intervention on total GWG for either ITT^n^ (−0.28 kg, 95% CI –1.43 to 0.87, *P*=.63) data or PPD^o^ (−0.65 kg, 95% CI –1.89 to 0.67, *P*=.34). 7 BCTs^p^ were common to all effective interventions. Effective interventions averaged over twice as many BCTs from goals and planning, and feedback and monitoring domains as ineffective ones. Positive association between high engagement with key BCTs and greater intervention success. Interventions using proactive messaging and feedback appeared to have more engagement.
Iyawa et al [75]	Systematic review	All pregnant women	1/18 (6)	Use of mobile apps during pregnancy points toward positive impact on pregnancy and health service delivery. Mobile apps can facilitate communication between pregnant women and HCPs^q^ despite distance, making them a suitable option for patients in areas with less access to HCPs and medical facilities.
Leonard et al [37]	Meta-analysis	All pregnant women	6/21 (29)	Women in technology-supported IG had significantly lower mean GWG than CG (mean GWG −1.18, Cohen *d*=0.23). Relatively small effects may be improved by intervention characteristics such as delivery mode, type of technology, and frequency prescribed.
Barroso et al [41]	Scoping review	Overweight & obese pregnant women	1/8 (13)	Out of 8 identified trials, 4 had lifestyle interventions that were effective in improving GWG. Effective interventions were intensive, included in-person sessions, started early-to-mid pregnancy, and lasted remaining pregnancy duration. Lifestyle coaches trained in behavior change and motivational interviewing can facilitate in-person sessions, helping set small goals and use self-monitoring strategies, and providing feedback.
Henriksson et al [83]	Secondary analysis RCT data	All healthy pregnant women	—	Greater number of registrations within app was associated with lower GWG and improved diet quality. Results were mainly attributable to the number of PA registrations. Number of app sessions and page views were not associated with GWG, diet quality, and PA.
Wu et al [70]	Meta-analysis	Overweight and obese pregnant women	2/23 (9)	Compared with CAU^r^, women with PA, diet, and combined diet+PA interventions all gained less during pregnancy (mean GWG –1.98kg, 95% CI –3.50 to –0.47), –1.95 kg (95% CI –3.19 to –0.71), and –1.21 kg (95% CI –1.92 to –0.50), respectively).
Raab et al [79]	Scoping review	All pregnant women	13/97 (13)	In 7 of 18 included (pilot) RCTs, rates of eGWG or total GWG could be reduced by intervention. Effectiveness and implementability of app-supported interventions have yet to be determined. Identifying most beneficial app features and intervention components is challenging. Consistent and comprehensive intervention and outcome reporting is needed.

a RCT: randomized controlled trial.

b Not applicable.

c CG: control group.

d IG: intervention group.

e GWG: gestational weight gain.

f eGWG: excessive gestational weight gain.

g GDM: gestational diabetes mellitus.

h IPD: individual participant data

i PA: physical activity.

j RR: relative risk.

k mHealth: mobile health.

l PPWR: postpartum weight retention.

m PPWL: postpartum weight loss.

n ITT: intention-to-treat.

o PPD: per-protocol data.

p BCT: behavior change technique.

q HCP: health care professional.

r CAU: care as usual.

#### Intervention Characteristics of Primary Data Articles

The 23 primary data articles analyzed 22 distinct interventions on which we focus in this section. In general, studies often provided insufficient detail in intervention descriptions, representing a gap in reporting practices that complicated data extraction and interpretation of successful design and implementation features. Despite this, we were able to extract the following information.

Most interventions targeted pregnant women in their first or second trimester who were overweight or obese, had singleton pregnancies, and no medical conditions or pregnancy complications that could affect metabolism or body weight. Exceptions included 2 studies (9%) that also included women with normal weight [34,67], 1 (5%) focusing on sedentary women [61], and 1 (5%) targeting women who had entered their weight gain in a specific app [66].

Recruitment was mostly done through prenatal clinics (n=9), followed by (university) hospital obstetric units (n=6), maternity units (n=3), and social media platforms (n=4). Most interventions used the 2009 IOM GWG guidelines (n=20, 91%). Regarding lifestyle, most interventions targeted both PA and diet (n=18, 82%), while 2 interventions (9%) focused solely on PA [55,57], and 2 (9%) on diet only [54,59].

#### Theoretical Foundation

Interventions were underpinned by a myriad of behavioral and social theories (Table 3), with social cognitive theory being most prevalent (n=11, 50%). Notably, 4 interventions (18%) used more than 1 theory [9,34,57,60], while 5 interventions (23%) provided no identifiable theoretical basis.

**Table 3.** Theoretical foundations of the interventions.

Theory	Number of studies	Study reference
Social cognitive theory	11	Ferrara et al [9], Koivuniemi et al [33], Sandborg et al [35], Pollak et al [51], Willcox et al [53], Thomas et al [57], Waring et al [58], Herring et al [60], Smith et al [61], Gonzalez-Plaza et al [64], Chen et al [65]
Control theory	1	Soltani et al [52]
Social ecological model	1	Herring et al [60]
Integrative model of behavior prediction	1	Olson et al [34]
Behavior model for persuasive design	1	Olson et al [34]
Theory of planned behavior	1	Downs et al [56]
Transtheoretical model of change	4	Ferrara et al [9], Redman et al [31], Thomas et al [57], Souza et al [67]
Self-determination theory	1	Darvall et al [55]
No theory mentioned	5	Feng et al [32], Li et al [54], Mattson et al [59], Van Horn et al [62], Litman et al [66]

#### Timing

Intervention timing was relatively consistent across studies, with most starting between gestational weeks 10 and 16 on average. Specifically, 4 interventions started before 14 weeks of gestation (range 6‐13, 18%) [31,32,56,57]; 13 interventions started before 28 weeks of gestation (range 8‐28, 59%) [9,33-35,53,55,59-61,64-67]; and 5 interventions started between 14 and 28 weeks of gestation (range 14‐20, 23%) [51,52,54,58,62].

#### Duration

The average intervention duration was 21.1 (SD 7.3, range 6‐29, median 24) weeks. Interventions starting in trimester 1 (<14 wk gestation) lasted about 7 weeks longer (average 28, SD 1.0 wk) than those starting in either trimester 1 or 2 combined (<28 wk gestation; average 21, SD 6.9 wk) and about 11 week longer than those starting in trimester 2 (≥14 wk and <28 wk gestation; average 16 wk, SD 1.5 wk).

#### Frequency

Frequency patterns varied considerably across studies, ranging from on-demand content access to structured daily check-ins combined with in-person sessions and some interventions combining multiple formats (detailed descriptions in Multimedia Appendix 4).

#### Delivery Mode

Most interventions were digital-only (n=13, 59%) [32-35,51,54,58-60,64-67], with 7 incorporating mobile health (mHealth) tools, such as smart watches, smart scales, or pedometers [34,35,59,64-67]. The other 9 interventions used digital-mixed formats (41%) [9,31,52,53,55-57,61,62], of which 3 also used mHealth tools [55-57].

#### BCTs

Across all interventions, a total of 227 BCTs were identified, averaging 9 BCTs per intervention (range 3‐18). The majority of BCTs originated from the clusters “goals and planning,” “feedback and monitoring,” and “social support.” The 3 most common individual BCTs were “self-monitoring of behavior” (code 2.3), “goal setting (behavior)” (code 1.1), and “self-monitoring of outcome(s) of behavior” (code 2.4). A full taxonomy of BCTs and their frequency is presented in Table 4.

**Table 4.** Frequency of BCTs^a^ across the 22 interventions, with proportions expressed as percentages in parentheses.

Cluster and code	BCT	Count, n (%)
Goals and planning		
1.1	Goal setting (behavior)	18 (82)
1.2	Problem solving	11 (50)
1.3	Goal setting (outcome)	12 (55)
1.4	Action planning	6 (27)
1.5	Review behavior goals	8 (36)
1.6	Discrepancy between current behavior and goal	5 (23)
1.7	Review outcome goal(s)	6 (27)
1.8	Behavioral contract	0 (0)
1.9	Commitment	0 (0)
Feedback and monitoring		
2.1	Monitoring of behavior by others without feedback	0 (0)
2.2	Feedback on behavior	12 (55)
2.3	Self-monitoring of behavior	19 (86)
2.4	Self-monitoring of outcomes of behavior	16 (73)
2.5	Monitoring outcome(s) of behavior by others without feedback	1 (5)
2.6	Biofeedback	1 (5)
2.7	Feedback on outcomes of behavior	10 (45)
Social support		
3.1	Social support (unspecified)	11 (50)
3.2	Social support (practical)	4 (18)
3.3	Social support (emotional)	4 (18)
Shaping knowledge		
4.1	Instruction on how to perform a behavior	15 (68)
4.2	Information about antecedents	3 (14)
4.3	Reattribution	0 (0)
4.4	Behavioral experiments	0 (0)
Natural consequences		
5.1	Information about health consequences	13 (59)
5.2	Salience of consequences	0 (0)
5.3	Information about social and environmental consequences	0 (0)
5.4	Monitoring of emotional consequences	0 (0)
5.5	Anticipated regret	0 (0)
5.6	Information about emotional consequences	1 (5)
Comparison of behavior		
6.1	Demonstration of the behavior	4 (18)
6.2	Social comparison	3 (14)
6.3	Information about others’ approval	0 (0)
Associations		
7.1	Prompts or cues	13 (59)
7.2	Cue signaling reward	0 (0)
7.3	Reduce prompts or cues	0 (0)
7.4	Remove access to the reward	0 (0)
7.5	Remove aversive stimulus	0 (0)
7.6	Satiation	0 (0)
7.7	Exposure	0 (0)
7.8	Associative learning	0 (0)
Repetition and substitution		
8.1	Behavioral practice or rehearsal	2 (9)
8.2	Behavior substitution	0 (0)
8.3	Habit formation	0 (0)
8.4	Habit reversal	0 (0)
8.5	Overcorrection	0 (0)
8.6	Generalization of a target behavior	0 (0)
8.7	Graded tasks	0 (0)
Comparison of outcomes		
9.1	Credible source	13 (59)
9.2	Pros and cons	0 (0)
9.3	Comparative imagining of future outcomes	0 (0)
Reward and threat		
10.1	Material incentive (behavior)	2 (9)
10.2	Material reward (behavior)	1 (5)
10.3	Nonspecific reward	1 (5)
10.4	Social reward	3 (14)
10.5	Social incentive	0 (0)
10.6	Nonspecific incentive	0 (0)
10.7	Self-incentive	0 (0)
10.8	Incentive (outcome)	1 (5)
10.9	Self-reward	2 (9)
10.10	Reward (outcome)	1 (5)
10.11	Future punishment	0 (0)
Regulation		
11.1	Pharmacological support	0 (0)
11.2	Reduce negative emotions	0 (0)
11.3	Conserving mental resources	0 (0)
11.4	Paradoxical instructions	0 (0)
Antecedents		
12.1	Restructuring the physical environment	2 (9)
12.2	Restructuring the social environment	0 (0)
12.3	Avoidance or reducing exposure to cues for the behavior	1 (5)
12.4	Distraction	0 (0)
12.5	Adding objects to the environment	0 (0)
12.6	Body changes	0 (0)
Identity		
13.1	Identification of self as role model	0 (0)
13.2	Framing or reframing	0 (0)
13.3	Incompatible beliefs	0 (0)
13.4	Valued self-identity	0 (0)
13.5	Identity associated with changed behavior	0 (0)
Scheduled consequences		
14.1	Behavior cost	0 (0)
14.2	Punishment	0 (0)
14.3	Remove reward	0 (0)
14.4	Reward approximation	0 (0)
14.5	Rewarding completion	0 (0)
14.6	Situation-specific reward	0 (0)
14.7	Reward incompatible behavior	0 (0)
14.8	Reward alternative behavior	0 (0)
14.9	Reduce reward frequency	0 (0)
14.10	Remove punishment	0 (0)
Self-belief		
15.1	Verbal persuasion about capability	1 (5)
15.2	Mental rehearsal of successful performance	0 (0)
15.3	Focus on past success	1 (5)
15.4	Self-talk	0 (0)
Covert learning		
16.1	Imaginary punishment	0 (0)
16.2	Imaginary reward	0 (0)
16.3	Vicarious consequences	0 (0)

a BCT: behavior change techniques.

### Outcomes

Digital lifestyle interventions showed varied outcomes for healthy GWG across both primary and secondary studies, with certain design and implementation features appearing more frequently in interventions that achieved their intended outcomes. Among 12 primary data articles coded for achieving their intended outcomes, 7 (58%) reported beneficial effects of digital lifestyle interventions on healthy GWG [9,31,32,60,62,64,66]. These interventions resulted in lower total mean or median GWG (average Cohen *d*=0.42; range 0.33-0.55) [9,32,60,62,64], reduced weekly rate of GWG [9,64], reduced risk for excessive GWG [66], and improved adherence to GWG guidelines [9,31,60,62]. The remaining 5 studies (42%) did not achieve their intended outcome [34,35,61,65,67]. Although the 10 pilot RCTs were underpowered for definitive conclusions and thus not coded for achieving their intended outcomes (refer to Methods section), most reported beneficial trends for healthy GWG (n=7, 70%) [51-56,59] and 2 (20%) described better adherence to IOM guidelines [52,54].

Secondary data articles reinforce these findings. Of the 9 meta-analyses, 7 (78%) found that interventions achieved their intended outcomes [37,40,69-73], with pregnant women in intervention groups gaining an average of 1.4 (SD 0.71) kg less than control groups. All 4 secondary analyses of RCT data reported positive outcomes for digital lifestyle interventions, including effects for subgroups of participants with mid-high income who consistently used digital interventions (average −2.1, SD 0.26 kg) [81,82], associations between increased digital engagement with lower GWG [83], and differential effects by BMI status [36]. All 5 systematic reviews found digital lifestyle interventions promising for healthy GWG [74-78]. The 3 scoping reviews similarly found that approximately half of the digital interventions included improved healthy GWG outcomes [41,79,80].

### Design and Implementation Features Linked to Achieving Outcomes

**Figure 4.**
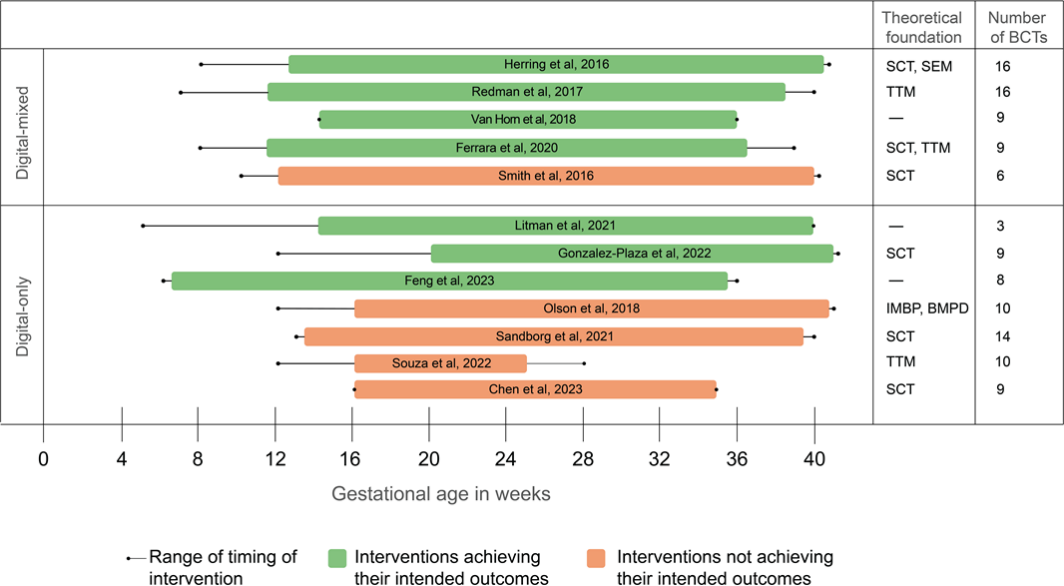
Summary overview of design and implementation features in the 12 interventions [9,31,32,34,35,60-62,64-67] coded for achieving their intended outcomes. BCT: behavior change technique; BMPD: behavior model for persuasive design; IMBP: integrative model for behavior prediction; SEM: social ecological model; SCT: social cognitive theory; TTM: transtheoretical model of change.

Figure 4 represents a summary of design and implementation features of the 12 interventions that we coded for achieving their intended outcomes, followed by a description of the main findings per feature.

#### Theoretical Foundation

The relationship between theoretical grounding and intervention outcomes was inconsistent across studies. In the primary data articles, social cognitive theory was the most frequently used theoretical framework, used in interventions with varying outcomes. Among the 7 interventions that achieved their stated objectives, 2 were based on 2 theories (combining the social cognitive theory and the social ecological model [60] [14%] or the transtheoretical model of change [9] [14%]). Notably, 3 interventions (43%) that met their objectives reported no explicit theoretical foundation [32,62,66]. Of the 5 interventions that did not achieve their stated goals, 3 (60%) were based on social cognitive theory [35,61,65], and 1 (20%) combined the integrative model of behavior prediction with the behavior model for persuasive design [34]. Secondary data articles did not examine theoretical foundations as determinants of intervention outcomes.

#### Timing

Primary data articles showed that interventions achieving their stated objectives were initiated earlier in pregnancy (average 12, SD 4.1 wk, range 6‐28 wk) compared with those that did not (average 14, SD 1.9 wk, range 10‐20 wk, Figure 4). This pattern was consistent with findings from secondary data sources reporting that digital interventions meeting their outcomes typically commenced in early-to-mid pregnancy [40,41,70,79,80].

#### Duration

Intervention duration varied between studies with different outcomes, with interventions that achieved their stated objectives having longer durations (mean 25.5, SD 3.3 wk, range 21‐29 wk, median 27 wk) compared with those that did not (mean 21.4, SD 6.9 wk, range 9‐28 wk, median 25 wk). Secondary data sources described similar patterns, with Barroso et al [41] noting that digital interventions meeting their outcomes typically spanned the remaining pregnancy duration. Hutchesson et al [80] identified intervention duration and intensity as areas requiring further research.

#### Frequency

As stated earlier, the frequency of intervention delivery showed large differences between interventions. There were no clear differences in delivery cadence between interventions that met their intended outcomes and those that did not. Secondary data articles also reported that frequency did not have a consistent impact on interventions achieving their objectives [37,38,40,71].

#### Delivery Mode

Primary data articles revealed variation in outcomes between delivery modes, with 4 digital-mixed interventions (57%) achieving their intended outcomes [9,31,60,62] and 3 digital-only ones (43%) [32,64,66]. Among the interventions that did not meet their objectives, 1 was digital-mixed (20%) [61] and 4 were digital-only (80%) [34,35,65,67]. Secondary data sources corroborate these patterns, with Barroso et al [41] noting that digital interventions attaining their goals typically included frequent touch points with well-trained lifestyle coaches through in-person sessions. Hutchesson et al [80] similarly identified delivery modes in interventions and involvement of health care providers as areas for further investigation.

#### BCTs

Primary data articles indicated that interventions meeting their objectives used the same number of BCTs (average 10, SD 4.3, range 3‐16) compared with those that did not (average 10, SD 2.6, range 6‐14). Table 5 maps the BCTs used across interventions that achieved their stated objectives and those that did not meet their goals. BCTs used commonly (≥70%) across both intervention categories were goal setting (behavior) and self-monitoring of outcome(s) of behavior.

**Table 5.** List of behavior change techniques used in interventions achieving (n=7) and not achieving (n=5) intended outcomes.

Code	BCT^a^	Interventions achieving intended outcomes, n (%)	Interventions not achieving intended outcomes, n (%)	Total
1.1	Goal setting (behavior)	5 (71^b^)	4 (80^b^)	9
1.2	Problem solving	2 (29)	3 (60^c^)	5
1.3	Goal setting (outcome)	5 (71^b^^,c^)	2 (40)	7
1.4	Action planning	1 (14)	1 (20)	2
1.5	Review behavior goal(s)	2 (29)	1 (20)	3
1.6	Discrepancy between current behavior and goal	3 (43^c^)	0 (0)	3
1.7	Review outcome goal(s)	1 (14)	1 (20)	2
2.2	Feedback on behavior	3 (43)	2 (40)	5
2.3	Self-monitoring of behavior	6 (86^b^^,c^)	3 (60)	9
2.4	Self-monitoring of outcome(s) of behavior	6 (86^b^)	4 (80^b^)	10
2.5	Monitoring outcome(s) of behavior by others without feedback	0 (0)	1 (20)	1
2.6	Biofeedback	0 (0)	1 (20)	1
2.7	Feedback on outcome(s) of behavior	4 (57)	3 (60)	7
3.1	Social support (unspecified)	5 (71^b^^,c^)	1 (20)	6
3.2	Social support (practical)	1 (14)	1 (20)	2
3.3	Social support (emotional)	1 (14)	2 (40^c^)	3
4.1	Instruction on how to perform a behavior	5 (71^b^)	3 (60)	8
4.2	Information about antecedents	1 (14)	1 (20)	2
5.1	Information about health consequences	5 (71^b^)	3 (60)	8
6.1	Demonstration of the behavior	1 (14)	1 (20)	2
6.2	Social comparison	1 (14)	0 (0)	1
7.1	Prompts or cues	4 (57)	4 (80^b^)	8
8.1	Behavioral practice or rehearsal	0 (0)	1 (20)	1
9.1	Credible source	5 (71^b^^,c^)	2 (40)	7
10.1	Material incentive (behavior)	0 (0)	1 (20)	1
10.10	Reward (outcome)	0 (0)	1 (20)	1
10.2	Material reward (behavior)	1 (14)	0 (0)	1
10.3	Nonspecific reward	0 (0)	1 (20)	1
10.4	Social reward	1 (14)	1 (20)	2
10.9	Self-reward	1 (14)	0 (0)	1

a BCT: behavior change technique.

b BCTs that were used often, that is, in ≥70% of interventions (also refer to Multimedia Appendix 3);

c BCTs that were used more, that is, ≥25% in successful interventions as compared with an unsuccessful one or vice versa (also refer to Multimedia Appendix 3).

In total, 5 BCTs showed notable differences in usage patterns between intervention categories, appearing ≥25% more frequently in interventions that achieved their objectives: goal setting (outcome; 71% vs 40%), discrepancy between current behavior and goal (43% vs 0%), self-monitoring of behavior (86% vs 60%), social support (unspecified; 71% vs 20%), and credible source (71% vs 40%). Conversely, interventions that did not meet their objectives used problem solving (60% vs 29%) and social support (emotional; 40% vs 14%) ≥25% more frequently than those that achieved their goals.

Interestingly, with respect to delivery modes, we observed different patterns in the number of BCTs used in interventions meeting their objective: digital-mixed interventions (n=4, 57%) used 13 BCTs, while digital-only ones used 7 (n=3, 43%). Most commonly used BCTs in successful digital-mixed interventions were goal setting (behavior and outcome) and self-monitoring of behavior. In contrast, successful digital-only interventions primarily relied on self-monitoring of outcome(s) of behavior, information about health consequences, and a credible source.

From the secondary data articles, only the meta-analysis by Rhodes et al [68] examined BCTs in relation to intervention outcomes, reporting an average of 9 BCTs per intervention and confirming that interventions meeting their objectives used twice as many BCTs from the groups “goals and planning” and “feedback and monitoring” categories compared with interventions that did not. They identified 7 BCTs commonly present across interventions that attained their goal: goal setting (behavior), problem solving, review of behavior goals, feedback on behavior, social support, information about health consequences, and information about emotional consequences. Notably, review of behavior goal(s) was observed exclusively in interventions achieving their objectives, with the authors concluding that interactivity is crucial for driving engagement and improving intervention success. A secondary analysis of RCT data [36] described BMI-specific patterns for goal setting, noting beneficial associations for women with normal BMI but adverse associations for those with higher BMI.

## Discussion

### Evidence Landscape of Digital Lifestyle Interventions for Healthy GWG

Digital lifestyle interventions alongside usual care show potential for supporting healthy lifestyles and GWG management among pregnant women. This scoping review systematically mapped existing literature to identify key design and implementation features, examining theoretical frameworks, timing, duration, frequency, delivery modes, and BCTs. Our mapping of 44 articles (23 primary and 21 secondary data) revealed diverse approaches to digital lifestyle interventions targeting GWG management and highlighted gaps in current research. These findings provide insights into identifying research priorities and informing future intervention development.

Our initial research question focused on the scope and nature of evidence regarding digital lifestyle interventions for GWG. The primary data articles revealed diverse patterns in reported outcomes. Among the 23 primary studies reviewed [9,31-35,51-67], 12 were coded for success [9,31,32,34,35,60-62,64-67] with 7 achieving their intended outcomes [9,31,32,60,62,64,66]. In addition, 7 of the 10 pilot studies [33,51-59], although underpowered, indicated positive trends [51-56,59]. Notably, several studies that did not report improvements in GWG did document benefits such as increased PA and improved dietary quality [35,61,67]. The secondary data articles, particularly meta-analyses, commonly reported that digital interventions supported GWG, although the reported impacts were described as modest. These findings are consistent with broader literature suggesting potential benefits of digital lifestyle interventions for GWG [68].

The substantial body of secondary literature identified in this review indicates ongoing interest in synthesizing evidence on digital lifestyle interventions and their components. This is also reflected in the temporal analysis of the systematic reviews in our selection that revealed an evolving research focus and improvements in the quality of digital lifestyle interventions. Early work by O’Brien et al [74] in 2014 concluded that digital interventions hold potential for complementing traditional health care models but emphasized the low quality and quantity of the interventions available. By 2018, Overdijkink et al [76] reported that app interventions had good overall usability and efficacy, although high dropout rates for several apps persisted. Mertens et al [77] in 2019 confirmed that the quality of interventions improved, but challenges with accessibility and engagement with target populations remained. Subsequently, Hussain et al [78] reinforced the potential of digital interventions for managing healthy GWG but highlighted the continued heterogeneity of studies and their generally small sample sizes. They also called for cost-effectiveness research. In 2021, Iyawa et al [75] reported that apps could positively impact self-management, such as GWG, during pregnancy while noting gaps in longitudinal studies and research in low- and middle-income countries.

The continued heterogeneity of interventions described by Hussain et al [78] was also evident in the primary data articles we studied, with differences in study populations (such as participants from different prepregnancy BMI categories), intervention duration, intensity, and delivery modes. This diversity was explicitly acknowledged in several meta-analyses as a challenge for evidence synthesis [37,40,68,69,71]. In addition, studies used varied approaches to outcome measurement, with some focusing on adherence to IOM guidelines [9,31,60,62], others on the total average [9,31,60,62,63] or median GWG [32,64], weekly weight gain rates [9], patterns of intervention engagement in relation to weight outcomes [66], or any combination of these. This heterogeneity in both intervention design and outcome measurement approaches underscores the complexity of this research field. It points to areas where greater methodological consistency could benefit future research synthesis efforts.

### Patterns in Key Design and Implementation Features

#### Overview

Our second research question explored the key components and characteristics of digital lifestyle interventions for healthy GWG. This exploration involved mapping intervention components and examining patterns across different types of interventions to identify commonly reported features. We examined intervention theoretical foundations, timing, duration, frequency, delivery modes, and BCTs.

A significant gap emerged regarding the comprehensiveness of intervention descriptions across the reviewed articles. Many lacked sufficient detail for reliable interpretation of components and potential research replication. This finding was reinforced by our secondary data articles, which similarly identified gaps in reporting on interventions and their outcomes as a key limitation in the field. These documentation limitations align with previous research highlighting challenges in comparing interventions and synthesizing evidence about their characteristics [42,45,46,68,69,71]. Despite the publication of the Behavior Change Technique Taxonomy in 2013 [44], and the fact that included studies were published afterwards, the majority have not incorporated these standardized reporting guidelines. Despite these limitations, we identified tentative patterns between design features and intervention outcomes, discussed below in relation to existing literature.

#### Theoretical Foundations

Regarding theoretical foundations, we found that this feature did not appear to differentiate between interventions that achieved their intended outcomes and those that did not: both types of interventions commonly drew upon social cognitive theory. Potential explanations include inappropriate selection or poor application of the theory for a given intervention context [84]. Notably, 5 interventions in our review lacked a clearly defined theoretical foundation, with 3 of these reporting achievement of their intended outcomes. This pattern is noteworthy given available literature suggesting that theory-driven interventions are more likely to be successful [84,85]. Possible explanations include that theoretical frameworks were applied but not explicitly reported, or that intervention developers focused on implementing specific BCTs rather than grounding their approach in a central theory.

#### Timing and Duration

Earlier intervention initiation and longer duration were more commonly observed in interventions that achieved their stated objectives. This aligns with the understanding that weight gain accelerates in the second half of pregnancy, necessitating early intervention [66,86]. None of the interventions in our review commenced in the third trimester, which aligns with this rationale.

#### Frequency

No clear patterns emerged between contact frequency and intervention outcomes. Interventions showed substantial heterogeneity in contact points with providers and participant control over interaction with intervention components. Reasons for these differences likely stem from theory-based choices, for example, offering more frequent but less personalized elements versus less frequent but more tailored ones.

#### Delivery Mode

Digital-mixed interventions were more often associated with success than digital-only formats. As such, in-person components, especially those involving health care provider endorsement, may enhance credibility and behavior change, which is in line with existing research. However, as Rhodes et al [68] observed, methodological heterogeneity between interventions may have limited the ability to detect consistent patterns of association with intervention outcomes. Therefore, studies in which a comparison is made between delivering the same intervention through digital-mixed and digital-only means, such as that by Redman et al [31] that offers an interesting venue for future research.

#### BCTs

BCTs related to the categories goals and planning, and feedback and monitoring were frequently associated with successful interventions. While previous research by Webb et al [87] and Rhodes et al [68] suggested that a higher number of BCTs may enhance effectiveness, our review did not find a consistent relationship between the number of BCTs and intervention success. This discrepancy may reflect differences in scope: whereas both Webb et al and Rhodes et al examined digital-only interventions, our review included both digital-only and digital-mixed delivery. Despite this, the average number of BCTs per intervention in our review (average 9) matched that reported by Rhodes et al [68], suggesting some consistency in usage patterns.

More importantly, our analysis highlighted that specific BCTs, rather than the total number, were more commonly associated with successful outcomes. These included: goal setting (outcome), discrepancy between current behavior and goal, self-monitoring of behavior, social support (unspecified), and a credible source. These findings are supported by broader literature on diet and PA interventions, in which interventions combining self-monitoring with at least one other technique from control theory [88] – in this case, goal setting (outcome) – were more commonly associated with achieving intended outcomes [89].

When examining delivery modes, we observed that digital-mixed interventions used more BCTs than digital-only ones. This may suggest that digital-only interventions can be effective with fewer, well-targeted BCTs – an area that warrants further research, particularly given the challenges of adapting certain BCTs to digital formats [90].

#### In Summary

Summarizing, our review identified several implementation and design features that may contribute to digital lifestyle interventions for GWG in achieving their intended goals. While theoretical foundation and frequency of intervention delivery were not commonly associated with success, interventions that started earlier in pregnancy, lasted longer, and combined digital with nondigital components were more likely to achieve beneficial outcomes. In addition, the BCTs’ goal setting (outcome), discrepancy between current behavior and goal, self-monitoring of behavior, social support (unspecified), and credible source were commonly present across interventions accomplishing their intended purpose.

### Limitations and Strengths

This review has several limitations. First, we excluded articles involving high-risk pregnancies. Given the greater health stakes in such populations, it is possible that digital lifestyle interventions may have different levels of impact among at-risk women. Future research should explore whether outcomes differ between normal- versus high-risk pregnancies.

A second limitation is the inability to control for variations in usual care across different localities, which can vary substantially between health care settings. This may influence women’s opportunities, motivations, and capabilities to engage in healthy lifestyle behaviors. Consequently, the functional gap that a digital lifestyle intervention needs to address may differ depending on this local health care context.

Third, most included studies were conducted in high-income countries, mainly the United States. This limits the generalizability of our findings to low- and middle-income countries, where digital interventions may be especially valuable due to limited access to maternity care.

In addition, our searches included only publications in the English language and those published until March 25, 2024. This may have excluded relevant studies in other languages or published more recently.

Despite these limitations, our review has several strengths. We included both digital-only and digital-mixed interventions, enabling comparative insights. Our structured and replicable literature search strategy ensured comprehensive coverage of the literature. By focusing on healthy (normal-risk) pregnant populations, we were able to draw clearer comparisons across studies.

Finally, we conducted an extensive analysis of key intervention and design features used across the included interventions, including BCTs. This addressed a gap in the literature, as only a few previous reviews have provided a detailed breakdown of which specific BCTs are commonly used in digital lifestyle interventions with both digital-mixed and digital-only delivery modes for GWG management in pregnant women. Our analysis, therefore, provides a foundation for understanding the current landscape of BCT implementation in this field and may inform future research directions and intervention development considerations. Therefore, we believe that despite the limitations of our review, our conclusions about the key design and implementation features contributing to interventions achieving their intended goals remain valid.

### Future Research

Based on our findings, we propose 5 key priorities for future research, which we outline below.

#### Healthy Lifestyles Versus GWG

First, digital lifestyle interventions reported modest differences in GWG between interventions that met their intended goals and those that did not (average reduction of 1.4, SD 0.71 kg). This is in line with Dodd et al [91], whose meta-analysis of 78 studies on nondigital lifestyle interventions found an average decrease in GWG of 1.1 (95% CI –1.46 to –0.74) kg in pregnant intervention participants compared with controls. In combination with previous research in nonpregnant populations suggesting that more weight loss is needed before health benefits start showing [92,93] and our findings that digital lifestyle interventions can positively affect lifestyles even without successful outcomes for healthy GWG [35,61,67], future studies should explore whether these behavioral changes, rather than GWG alone, are the primary drivers of improved pregnancy outcomes.

#### Engagement and Standardization

Second, more work on methodological aspects is required, such as differentiating between digital and behavioral engagement in longitudinal interventions. Digital engagement refers to users’ interaction with intervention components, while behavioral engagement describes the adoption of target health behaviors. Understanding their temporal relationship is crucial: interventions typically require initial digital engagement until users establish habits, after which behavioral engagement becomes more meaningful for assessing success. Measuring one type when intending to measure the other could lead to misleading conclusions about what contributes to achieving intended outcomes. It is important to note that long-term engagement—whether digital or behavioral—may not always be necessary for intervention success [90]. Rather than pursuing maximum engagement, future research should focus on identifying “effective engagement” to achieve healthy GWG. In addition, broader use of the BCT taxonomy [44] and clearer reporting of implementation strategies would enhance comparability and replication.

#### Intervention Development

Third, concerning intervention development, a better understanding of individual BCTs and their implementation is needed. Our findings reveal an overlap between the most prominent BCTs in both effective and ineffective interventions, suggesting that BCT implementation methods may influence outcomes differently than the techniques themselves. For instance, social support can be delivered through forums, group settings, or peer-to-peer pairs, each with potentially different characteristics. Future research could explore BCT implementation approaches and their underlying mechanisms of action.

Another consideration involves recent technological trends in intervention development. As most pregnancy apps are developed by private companies [94], their features reflect current technological capabilities, including comprehensive data collection, wearable integration, and algorithm-driven coaching [95,96]. While these advances may enhance accessibility and personalization, they introduce important ethical considerations [97,98]. Particularly, personalization raises concerns about user autonomy [99] and trustworthiness [100], especially as intervention rules change through usage, potentially making one-time informed consent insufficient. In addition, pregnant women may place varying levels of trust in these systems despite potentially limited understanding of their capabilities [100]. Users may end up over- or undertrusting the system, both of which come at a cost [101]. Future research could explore how ethical considerations might be integrated into intervention development from the start, with attention to meaningful and ethical personalization.

#### Collaboration Between Academia and Industry

Fourth, we advocate enhanced collaboration between academia and industry in the research and development of digital lifestyle interventions for pregnant women. While our review identified many pilot RCTs, we observed a notable scarcity of follow-up studies scaling these initial findings. This gap may stem from the inherent challenges of transitioning from pilot studies to full-scale RCTs with technologically mature interventions within academic settings, where research typically progresses at a more measured pace and fewer resources for technological development are available. In contrast, industry partners operate on accelerated development cycles with direct access to emerging technologies. This difference in throughput time becomes particularly relevant given that while most published research originates from academic institutions, the majority of pregnancy-related apps in use are developed by private technology companies [94]. Strategic partnerships between these sectors could combine academia’s methodological rigor with industry’s technological agility, accelerating the translation of evidence-based interventions into widely accessible tools while maintaining scientific integrity, to together improve the health behavior of pregnant women and their families.

#### Societal Aspects

Finally, while there is a widespread assumption among researchers and policymakers that digital interventions for pregnancy could significantly benefit health care desert communities, this hypothesis remains largely unexplored in empirical research [94,102]. This gap is particularly noteworthy because, due to their unique circumstances and limited alternative resources, individuals in health care deserts may actually demonstrate higher success rates with digital interventions in terms of increased capability and opportunity to engage with healthy behaviors. In our scoping review, only Iyawa et al [75] addressed this crucial aspect. Furthermore, the health care desert context emphasizes the importance of conducting rigorous cost-effectiveness analyses, comparing different intervention approaches. Such economic evaluations are essential for making informed decisions about resource allocation, particularly in areas where health care resources are already scarce. This economic perspective becomes especially relevant when considering the potential scalability and sustainability of digital lifestyle interventions in underserved communities [103].

### Conclusion

This scoping review examined the landscape of digital lifestyle interventions aimed at supporting healthy (GWG), with a focus on 6 key design and implementation features: theoretical foundation, intervention timing, duration, frequency, delivery modes, and BCTs. Our findings confirm that digital interventions hold promise for promoting healthy GWG. While theoretical underpinnings and frequency of delivery did not consistently predict success, interventions that began earlier in pregnancy and lasted longer were more likely to achieve beneficial outcomes. Digital-mixed delivery modes—those combining digital tools with in-person contact—appeared more effective than digital-only formats. Importantly, 5 BCTs emerged as more commonly used in successful interventions: goal setting (outcome), discrepancy between current behavior and goal, self-monitoring of behavior, social support (unspecified), and credible source.

These findings provide a foundation for designing more effective, evidence-based digital interventions to support maternal health. Future research should continue to refine these components, explore their implementation in diverse populations, and address gaps in reporting and standardization.

## Supplementary material

10.2196/71548 Multimedia Appendix 1 Eligibility criteria.

10.2196/71548 Multimedia Appendix 2 Search strings for each of the databases searched.

10.2196/71548 Multimedia Appendix 3 Data items included in the Data Extraction file.

10.2196/71548 Multimedia Appendix 4 Data extraction table.

10.2196/71548 Checklist 1 PRISMA-ScR checklist.

## Abbreviations

BCT: behavior change technique
GWG: gestational weight gain
IOM: Institute of Medicine
mHealth: mobile health
PA: physical activity
PRISMA-ScR: Preferred Reporting Items for Systematic reviews and Meta-Analyses extension for Scoping Reviews
RCT: randomized controlled trial
