# Supplementary material for: Digital Lifestyle Interventions to Support Healthy Gestational Weight Gain: Scoping Review
Source: J Med Internet Res. 2025 Nov 14;27:e71548. doi: 10.2196/71548 (PMC12617965; doi:10.2196/71548)
Supplement: Multimedia Appendix 1 [file jmir-v27-e71548-s001.pdf]

Multimedia Appendix 2: Eligibility criteria

Inclusion criteria	Exclusion criteria
Population: Pregnant women	EC1. Article is not available in full in English
Concept: Digital lifestyle interventions for managing gestational weight gain	EC2. Article is not based on human subject data (e.g., computer-generated data)
Article is published between 2014 and March 2024 (we did not look before 2014 since a preliminary search showed there to be no relevant articles before that year)	EC3. Article is a study protocol (we excluded these since there are no results yet)
Article is on primary or secondary data	EC4. Article is not related to pregnancy
	EC5. Article does not have GWG as primary or secondary outcome
	EC6. Article is not peer-reviewed (e.g., congress abstracts, commentaries, grey literature, etc.; we excluded these to increase quality of our selection)
	EC7. Article is a duplicate
	EC8. Article does not investigate effects of digital lifestyle interventions
	EC9. Article focuses on high-risk pregnancies or pregnancy conditions (e.g., PP, GDM; we excluded these to focus on general GWG management rather than condition-specific interventions)

Abbreviations: EC: Exclusion Criterium; GWG: Gestational Weight Gain; PP: Placenta Previa; GDM: Gestational Diabetes Mellitus
