# Supplementary material for: Digital Lifestyle Interventions to Support Healthy Gestational Weight Gain: Scoping Review
Source: J Med Internet Res. 2025 Nov 14;27:e71548. doi: 10.2196/71548 (PMC12617965; doi:10.2196/71548)
Supplement: Multimedia Appendix 2 [file jmir-v27-e71548-s002.pdf]

Multimedia Appendix 3: Search strings for each of the databases searched

Database	Exact search string used	Filter / delimiter
Pubmed	((Smartphone Application) OR (Smart-phone Application) OR (Smartphone App) OR (Mobile application) OR (mHealth)) AND gestational weight gain	Publication date: 10 years
Embase	((smartphone NEAR/1 app*) OR (mobile NEAR/1 app*) OR 'mhealth') AND 'gestational weight gain'	Publication years: 2014 - 2024
Cochrane	((smartphone NEXT app*) OR (Mobile NEXT app*) OR ("mHealth")) AND "gestational weight gain”	Date published in Cochrane library from March 2014 to March 2024<br>Word variations have been searched
Web of Science	ALL=(((Smartphone Application) OR (Smart-phone Application) OR (Smartphone App) OR (Mobile application) OR (mHealth)) AND gestational weight gain)	Publication date: 2014-Mar-26 to 2024-Mar-25
