# Supplementary material for: Digital Lifestyle Interventions to Support Healthy Gestational Weight Gain: Scoping Review
Source: J Med Internet Res. 2025 Nov 14;27:e71548. doi: 10.2196/71548 (PMC12617965; doi:10.2196/71548)
Supplement: Multimedia Appendix 3 [file jmir-v27-e71548-s003.pdf]

Multimedia Appendix 4: Data items included in the Data Extraction file

Data Item	Description
**For primary and secondary data articles**	
ID	Unique identifier we assigned to the article
Group code	Identifier for studies belonging to larger research study involving same intervention
Title	Title of the article
Author(s)	Author(s) of the article
Year	Year in which the article was published
Publication details	Details of journal in which article was published (title, volume, etc.)
Study type	Whether study was an RCT, systematic review, meta-analysis, etc.
Article type	Whether article was on primary or secondary data<br>Primary data articles: RCTs, pilot RCTs, intervention studies, real-world data studies<br>Secondary data articles: Meta-analyses, systematic reviews, scoping reviews, secondary data from RCTs
Goal	Main objective of the study
Results	Main outcomes of the study
Successful	Whether positive effect (i.e., in line with IOM guidelines) on healthy GWG was reported Yes /No<br>Positive effects defined as: (1) Lower total average or median GWG; (2) Greater adherence to IOM GWG guidelines; (3) Lower weekly rate of GWG; and (4) Lower risk of gaining excessive weight
**For primary data articles**	
IoM guidelines	Whether intervention was based on IOM guidelines Yes / No
Population details	Main inclusion criteria of study's population, e.g., age, health status[a]
Target group	Population targeted by intervention (e.g., overweight women)[a]

Sample size	Size of the population included in the study's analyses
Port of entry	How participants entered the study (e.g., through midwife, flyers, ...)
Country	Country where data were collected
Study year	Year(s) in which data were collected (i.e., not year of recruitment)
Study during COVID-19	Whether data were collected during the COVID-19 pandemic Yes / No
Description intervention	Main details of intervention
Theory	Theory or theories on which intervention was based
Lifestyle type	Whether intervention targeted diet, physical activity or both
Enrollment GA	Weeks / interval of gestation at which participants were recruited
Intervention duration	Duration of intervention in weeks<br>If not reported, difference between average GA in weeks at start and end of intervention was used. If starting GA was not provided, and no other specification was given, average GA at recruitment was assumed to represent start of intervention.<br>If end of intervention was reported to be 'at delivery' without further specification, we assumed standard average pregnancy duration of 40 weeks.
Timing of intervention in GA weeks	Week / interval of gestation at which intervention was started<br>If not reported, we assumed average GA as recruitment represented start of intervention
Timing of intervention in trimester	Initiation of intervention in trim 1, trim 2, or either trim 1 or 2
Frequency	Description of how often and in what way intervention components were delivered to participants, containing information on both contact frequency and intensity of use (if applicable).
Delivery medium	How intervention was delivered (e.g., app, in-person, booklet, etc.)
Digital-only/digital-mixed	Whether intervention was delivered digital-only or also non-digitally<br>Digital-only interventions relied exclusively on digital channels: app, SMS (text messaging), e-mail, social media, Web sites, Telehealth system, telephone calls[c]

	Digital-mixed Interventions included both digital channels and non-digital components: pen-and-paper materials, and face-to-face sessions<br>mHealth tools that did not deliver any part of the intervention were not counted as digital channel
BCTs used	Name and code of BCTs used in the study, according to BCCT v1[d]<br>To identify and assess BCTs, methods sections of included articles were reviewed, and any available supplementary materials were consulted. Then, BCTT v1 was employed. BCTs extracted from each article by one author were double-checked by another author to ensure accuracy.<br>BCTs used in ≥ 70% of the interventions were coded as 'often used'.<br>BCTs used ≥ 25% *more* in certain interventions as compared to others were coded as 'used more'.
Effect size	Size of reported effect (for non-pilot studies with a care as usual group)<br>If not given, Cohen's *d* was calculated by subtracting average total GWG in Kg in digital(-mixed) intervention group from average total GWG in Kg in care as usual group and dividing that by pooled standard deviation. In 1 case OR of exceeding IOM guidelines was calculated by dividing odds of exceeding guidelines in intervention group by odds of exceeding them in control group.
**For secondary data articles**	
Study selection / population details	Main study selection criteria for reviews or population details for articles on secondary analyses of RCT data
Target group	Pregnant population of interest for secondary data analyses[a]
#Articles included	Number of articles included in secondary data analyses
(Average) sample size	Samples size averaged over all included studies for reviews, or as reported in articles on secondary analyses of RCT data<br>One scoping review reported ranges of sample sizes (i.e., ≤ 100, ≥101-1000, or ≥1001). One systematic review reported median sample size. If not reported for meta-analyses, scoping reviews and systematic reviews, average sample size was calculated by dividing total number of participants included in each article by total number of studies included in that article.

Overlap primary articles	Overlap in numbers in primary studies included across secondary data articles
Time period	Time period from which articles were included[b]

[a]We only report on pregnant women, i.e., if a study also included a non-pregnant population, we do not report on that.

[b]We report on publication years of included studies, not on the period searched.

[c]We consider modern telephones digital media since they convert voice into digital signals

[d]Michie S, Richardson M, Johnston M, et al. The Behavior Change Technique Taxonomy (v1) of 93 Hierarchically Clustered Techniques: Building an International Consensus for the Reporting of Behavior Change Interventions. *Ann Behav Med*. 2013;46(1):81-95. doi:10.1007/s12160-013-9486-6

Abbreviations: RCT: Randomized Controlled Trial; GWG: Gestational Weight Gain; IOM: Institute of Medicine; trim: trimester; BCT: Behavior Change Technique; BCCT: Behavior Change Technique Taxonomy; OR: Odds Ratio.
