# Supplementary material for: Digital Lifestyle Interventions to Support Healthy Gestational Weight Gain: Scoping Review
Source: J Med Internet Res. 2025 Nov 14;27:e71548. doi: 10.2196/71548 (PMC12617965; doi:10.2196/71548)
Supplement: Multimedia Appendix 4 [file jmir-v27-e71548-s004.pdf]

Primary data articles

ID	Group code	Title	Author(s)	Year	Publication details	Study type	Article type	Goal	IoM guidelines	Population details	Target group	Sample size	Port of entry	Country	Study year	Study during COVID-19	Description intervention	Theory	Lifestyle type	Enrollment GA	Intervention duration	Timing of intervention in GA weeks	Timing of intervention in trimester	Frequency	Delivery medium	Digital-only / Digital-mixed	BCTs used	Results	Success-full
S16		Maternal obesity management using mobile technology: a feasibility study to evaluate a text messaging based complex intervention during pregnancy	Soltani H, Duxbury AMS, Arden MA, Dearden A, Furness PJ, Garland C.	2015	*Journal of Obesity* . 2015;2015:1-10. doi:https://doi.org/10.1155/2015/814830	Pilot RCT	Primary	To evaluate the feasibility of a text messaging-based complex intervention (MOMTech) designed to support obese women (BMI ≥ 30) with healthier lifestyles and limit GWG.	Yes	- GA: 8 - 10 weeks<br>- Age: ≥ 18 years<br>- BMI: > 30 kg/m^2<br>- Singleton pregnancy<br>- Read and understand English<br>- Exclusion: complications such as diabetes, hypertension, antepartum haemorrhage, unexplained fetal loss/stillbirth, psychiatric illness	Obese	14	Recruitment happened via maternity units in Doncaster Royal Infirmary Hospital	UK	unknown	No	As analysis focused on apropriateness of a text messaging based intervention, there was no control group. Authors compared routinely collected data in the intervention group with pregnant women who had declined to participate. The MOMTech intervention included 2 daily text messages, 4 appointments with a healthy lifestyle midwife, goal setting for diet and physical activity, and the use of self-monitoring diaries.	Control theory	combined diet & PA	8 to 10	25	14 to 16	trim 2	4 live sessions, 3 phone calls, daily SMS	SMS, face-to-face	Digital-mixed	1.1 Goal setting (behavior)<br>1.2 Problem solving<br>1.4 Action planning<br>1.5 Review behavior goals<br>2.2 Feedback on behavior<br>2.3 self-monitoring of behavior<br>2.7 Feedback on outcome(s) of behavior<br>5.1 Information about health consequences<br>6.2 social comparison<br>9.1 credible source<br>15.1 Verbal persuasion about capability	1) Intervention group had lower mean GWG than control group (6.65 kg vs 9.74 kg; not tested statistically)<br>2) Less women in the intervention group exceeded IOM guidelines (28% vs 50%; not tested statistically).	NA
S28		Web-based behavioral intervention increases maternal exercise but does not prevent excessive gestational weight gain in previously sedentary women	Smith, K., Lanningham-Foster, L., Welch, A., Campbell, C.	2016	*Journal of Physical Activity and Health* . 2016;13(6):587-593. doi:https://doi.org/10.1123/jpah.2015-0219	RCT	Primary	To determine if previously sedentary women utilizing a Web-based behavioral intervention designed to increase sustained PA would prevent excessive GWG.	Yes	- GA: 10-14 weeks<br>- Age: 18 - 45 years<br>- Women with history of participating in < 3 sessions of exercise for 30 minutes or more per week for at least 6 months before conception<br>- English-speaking<br>- Exclusion: history of GDM, HDP, chronic disease, BMI < 18,5 $kg.m^2$, condition influencing metabolism	Sedentary women	51	Participants were recruited by local prenatal clinics and a large partnering hospital	USA	2013-2014	No	Participants were divided into intervention and control groups. All participants accessed a Web site incorporating SCT principles. Control participants could view general recommendations on prenatal diet and PA, while intervention participants had additional access to PA goal-setting modules, problem-solving tools, a journal, a calendar, and a community forum. Maternal anthropometric data were collected twice, during two 3-week data collection periods, each occurring in a different trimester.	Social cognitive theory	combined diet & PA	10 to 14	28	10 to 14	trim 1 and 2	3 live contact points (only for measurements), on demand digital	Web site, pen-and-paper	Digital-mixed	1.1 Goal setting (behaviour)<br>1.2 Problem solving<br>2.3 Self-monitoring of behavior<br>3.1 Social support (unspecified)<br>5.1 Information about health consequences<br>9.1 Credible source (app provided by HCP)	1) Compared to CG, IG significantly increased sustained PA (+54 min on average; P<.05).<br>2) IG had higher mean GWG than control group (13.6 kg vs 11.2 kg; Cohen's d=.45)<br>3) Amount of activity performed by women in IG was not sufficient to prevent eGWG.	No
P44	R2	Effectiveness of SmartMoms, a Novel eHealth Intervention for Management of Gestational Weight Gain: Randomized Controlled Pilot Trial	Redman LM, Gilmore LA, Breaux J, et al.	2017	*JMIR mHealth and uHealth* . 2017;5(9):e133. doi:https://doi.org/10.2196/mhealth.8228	RCT	Primary	To decrease the proportion of women who exceed the Institute of Medicine (IOM) 2009 GWG guidelines.	Yes	- GA: ≤ 12 weeks<br>- Age: 18 - 40 years<br>- BMI: ≥ 25 kg/m^2<br>- Singleton pregnancy<br>- Exclusion: fetal anomaly, hypertension, (history of) psychotic or eating disorder, human immunodeficiency virus, diabetis, contraindications to excercise	Overweight & obese	54	Recruitment happened through community clinics	USA	2013-2015	No	Participants were divided into 3 groups: control, SmartMoms remote (delivered through mobile phone) and SmartMoms in-person (clinic-based setting). SmartMoms included a personalized dietary intake prescription, self-monitoring weight against a personalized weight graph, activity tracking with a pedometer, receipt of health information, and continuous personalized feedback from counselors.	Transtheoretical model of change	combined diet & PA	< 13	27	10 to 13	trim 1	18 bi-weekly lessons, continuous digital feedback, on demand digital	app, face-to-face	Digital-mixed	1.1 Goal setting (behavior)<br>1.2 Problem solving<br>1.3 Goal setting (outcome)<br>1.4 Action planning<br>1.5 Review behavior goals<br>1.6 Discrepancy between current behavior and goals<br>1.7 Review outcome goal(s)<br>2.2 Feedback on behavior<br>2.3 Self-monitoring of behviour<br>2.4 Self-monitoring of outcome<br>2.7 Feedback on outcomes of behavior<br>3.1 Social support (unspecific)<br>4.1 Instruction on how to perform behavior<br>5.1 Information about health consequences<br>9.1 Credible source<br>10.9 Self-reward	1) Both intervention groups together (in-person and remote) had lower overall GWG than the control group (9.2 kg vs 12.8 kg; *P* =.04).<br>2) The in-person intervention group gained less overall weight compared to the control grop (8.0 kg vs 12.8 kg; *P* =.04).<br>3) The remote intervention group gained less overall weight compared to the control group, but this was only a trend (10 kg vs 12.8 kg; *P* =.07).<br>4) The rate of GWG was lower in the in-person intervention group compared to the control group (0.31 kg/week vs 0.49 kg/week; *P* =.04), and comparable to the remote intervention group.<br>5) The proportion of women with excess GWG was significantly lower in both intervention groups compared to usual care (56% (*P* =.03) and 58% (*P* =.04; OR=.25) versus 86.4%, respectively).	Yes[a]
P3		A mobile health intervention promoting healthy gestational weight gain for women entering pregnancy at a high body mass index: the txt4two pilot randomised controlled trial	Willcox J, Wilkinson S, Lappas M, et al.	2017	*BJOG: An International Journal of Obstetrics & Gynaecology* . 2017;124(11):1718-1728. doi:https://doi.org/10.1111/1471-0528.14552	Pilot RCT	Primary	To determine the feasibility and effectiveness of an mHealth intervention promoting healthy diet, physical activity and gestational weight gain in pregnant women.	Yes	- GA: ≤12 weeks<br>- Age: ≥18<br>- BMI: ≥25 kg/m^2<br>- Singletons pregnancy<br>- Australia-based<br>- English speaking<br>- Exclusion: comorbidities requiring significant medical and/or dietary management, discontinuation of hospital care	Overweight & obese	91	Recruitment took place during first hospital antenatal visit to a university-affiliated maternity hospital in Melbourne, Australia.	Australia	2014-2015	No	Participants were divided into and intervention (txt4two) and a control group. Both groups received CAU, including brochures with advice on diet and PA. The intervention group additionally received a multi-modality delivered intervention including tailored text messages, access to a responsive information Web site viewable on mobile devices, video messages, and chat room interaction via Facebook. Intervention group participants received guidance from a trained researcher who educated them on nutrition, PA and GWG goals, and helped them track weight and set goals. They received 4-5 tailored text messages per week to help review their weight and behavioral goals.	Social cognitive theory	combined diet & PA	10 to 17	21	13 to 17	trim 1 and trim 2	4-5 SMS per week, 2 weekly Facebook posts, on demand digital	SMS, social media, Web site, pen-and-paper, face-to-face	Digital-mixed	1.1 Goal setting (behavior)<br>1.2 Problem solving<br>1.3 Goal setting (outcome)<br>1.4 Action planning<br>1.5 Review behavior goal(s)<br>2.2 Feedback on behavior<br>2.3 Self-monitoring of behavior<br>2.4 Self-monitoring of outcome(s) of behavior<br>3.1 Social support (unspecified)<br>4.1 Instruction on how to perform a behavior<br>5.1 Information about health consequences<br>5.6 Information about emotional consequences<br>6.2 Social comparison<br>7.1 Prompts/cues<br>9.1 Credible source<br>10.9 Self-reward<br>12.1 Restructuring the physical environment<br>15.3 Focus on past success	There was a significant difference in GWG between groups, with intervention group participants gaining 7.8 kg and the control group 9.7 kg (*P* =.04).	NA
P31		Dietary Approaches to Stop Hypertension Diet and Activity to Limit Gestational Weight: Maternal Offspring Metabolics Family Intervention Trial, a Technology Enhanced Randomized Trial	Van Horn L, Peaceman A, Kwasny M, et al.	2018	*American Journal of Preventive Medicine* . 2018;55(5):603-614. doi:https://doi.org/10.1016/j.amepre.2018.06.015	RCT	Primary	To investigate whether a calorie-controlled, Dietary Approach to Stop Hypertension (DASH)–type diet and lifestyle intervention guided by a Registered Dietitian Nutritionist coach using commercially available weight-loss technology could safely be applied to limit GWG and improve diet quality and physical activity.	No	- GA: <16 weeks<br>- Age: 18 - 45 years<br>- BMI: 25 - 40 kg/m^2<br>- Singleton pregnancy<br>- Fluent in English<br>- Exclusion: diabetes or HbA1c >6.5% measured prior to enrollment, in vitro fertilization, weight difference >15 lb relative to self-reported pre-pregnancy weight, substance abuse, smoking, plans to terminate pregnancy, or plans to move out of the area	Overweight & obese	280	Recruitment took place across ten obstetric practices that delivered patients at Northwestern Memorial Hospital, Chicago	USA	2012-2015	No	Participants were divided into an intervention and control group.The usual-care group received biweekly newsletters and links to publicly available maternity Web sites. The intervention group received dietitian-led Dietary Approaches to Stop Hypertension (DASH) diet and physical activity coaching through three individual and six group counseling sessions conducted via phone and webinar. A commercially available smartphone app was used for self-monitoring of diet and physical activity, with additional adherence support provided through telephone calls, text message prompts, and email reminders to encourage app usage and Web site engagement.	Not described	combined diet & PA	< 16	21	15	trim 2	9 remote live sessions, regular e-mails / SMS, on-demand digital, continuous monitoring (app / mHealth tools)	app, face-to-face	Digital-mixed	1.1 Goal setting (behavior)<br>1.2 Problem solving<br>1.3 Goal setting (outcome)<br>1.5 Review behavior goal(s)<br>2.2 Feedback on behavior<br>2.3 Self-monitoring of behavior<br>3.1 Social support (unspecified)<br>7.1 Prompt/Cues<br>9.1 Credible source	1) The intervention group gained significantly less weight that the control group (10 kg vs 12 kg; *P* =.02; Cohen's *d* =.33).<br>2) Less women in the intervention group exceeded the IOM guidelines (67% vs 84%, *P*= .004; OR=0.40).	Yes[a,b]
P71	R1	Mood and quality of life changes in pregnancy and postpartum and the effect of a behavioral intervention targeting excess gestational weight gain in women with overweight and obesity: a parallel-arm randomized controlled pilot trial	Altazan AD, Redman LM, Burton JH, Beyl RA, Cain LE, Sutton EF, Martin CK.	2019	*BMC pregnancy and childbirth.* 2019;19(1):50. doi:https://doi.org/10.1186/s12884-019-2196-8	RCT	Primary	1) To quantify changes in mental and physical quality of life and depressive symptoms across pregnancy and the postpartum period,.<br>2) To determine if gestational weight gain was associated with changes in mood and quality of life.<br>3) To assess the effect of a behavioral intervention targeting excessive gestational weight gain on mood and quality of life.	Yes	See P44	See P44	43	See P44	See P44	See P44	See P44	See P44	See P44	See P44	See P44	See P44	See P44	See P44	See P44	See P44	See P44	See P44	1) The proportion of women who had GWG exceeding IOM 2009 guidelines was 56.3% in the SmartMoms intervention and 81.8% in the Usual Care group (*P* =0.17; OR=0.23).<br>2) Women in the SmartMoms® intervention had less overall gestational weight gain as compared to the women in the Usual Care group (Usual Care: LS mean 12.8 kg, and SmartMoms intervention: LS mean 8.7 kg; *P* =0.03; Cohen's *d* =.3.33).	Yes[a,b]
P4		A Pedometer-Guided Physical Activity Intervention for Obese Pregnant Women (the Fit MUM Study): Randomized Feasibility Study	Darvall JN, Wang A, Nazeem MN, et al.	2020	*JMIR mHealth and uHealth* . 2020;8(5):e15112. doi:https://doi.org/10.2196/15112	Pilot RCT	Primary	To test a pedometer-based intervention to increase activity and reduce excessive GWG in pregnant women.	No	- GA: 12 - 16 weeks<br>- Age: ≥ years<br>- BMI ≥ 30 $kg.m^2$<br>- Exclusion: preeclampsia, multiple pregnancies, preterm rupture of membranes, incompetent cervix/cerclage, joint or muscle disorder sufficient to impair walking to a target of 10,000 steps daily	Obese	27	Patients were recruited when attending the antenatal clinic	Australia	2017-2018	No	Participants were divided into 3 groups: control, app intervention, and app + coach intervention. All patients were supplied with Fitbit Zip pedometer and instructed to wear it daily. The pedometer measured step counts and minutes each day spent at various activity levels. The display of the pedometer in the control group was obscured and could not be synced to their smartphones. Participants in the app and app-coach groups had the pedometer synced to their personal smartphone via the Fitbit app allowing automatic daily uploading of activity data. Participants in the app + coach group were also administered a behavioral change program consisting of 4 coaching sessions.	Self determination theory	physical activity	12 to 16	16	13 to 19	trim 1 and trim 2	1 live session, 3 phone calls, continuous monitoring (mHealth tools)	app, face-to-face, telephone, mHealth tools	Digital-mixed	1.1 Goal setting (behavior)<br>1.2 Problem solving<br>1.4 Action planning<br>1.5 Review behavior goal(s)<br>1.7 Review outcome goal(s)<br>2.3 Self-monitoring of behavior<br>2.4 Self-monitoring of outcome(s) of behavior<br>4.1 Instruction on how to perform a behavior<br>9.1 Credible source	There was no significant difference between groups in GWG: app group: -5.46 kg, *P* =.07; app-coach group:-0.40 kg, *P* =.89, both compared to control group.	NA
P11		A telehealth lifestyle intervention to reduce excess gestational weight gain in pregnant women with overweight or obesity (GLOW): a randomised, parallel-group, controlled trial	Ferrara A, Hedderson MM, Brown SD, et al.	2020	*The Lancet Diabetes & Endocrinology* . 2020;8(6):490-500. doi:https://doi.org/10.1016/S2213-8587(20)30107-8	RCT	Primary	To reduce excess GWG through a behavioural lifestyle intervention adapted from the Diabetes Prevention Program and delivered primarily by telehealth to be feasible in health-care delivery settings.	Yes	- GA: ≤12 weeks<br>- Age: ≥18<br>- BMI: 25 – 40 kg/m^2<br>- Singletons pregnancy<br>- US-based<br>- Exclusion: fertility-assisted pregnancy, bed rest, diabetes, current uncontrolled hypertension, thyroid disease, history of cardiovascular, cancer, lung or serious gastrointestinal disease, history of eating disorder or bariatric surgery, serious mental illness, recent history of mood or anxiety disorder, drug or alcohol use disorder, gestational diabetes	Overweight & obese	394	Recruitment took place during baseline clinic visit at Kaiser Permanente Northern California antenatal medical care	USA	2014-2018	No	Participants were divided into intervention and control groups. The intervention group received a core lifestyle program, which included in-person and telephone sessions focused on behavioral strategies to improve weight, diet, physical activity, and stress management. Dietitians delivered the intervention using motivational interviewing, social cognitive theory, and a phased approach to behavior change. The control group received standard antenatal care.	Transtheoretical model of change; social cognitive theory	combined diet & PA	< 14	24	8 to 15	trim 1 and trim 2	13 on-site or remote live sessions, on-demand telephone	pen-and-paper, face-to-face, telephone	Digital-mixed	1.1 Goal setting (behavior)<br>1.3 Goal setting (outcome)<br>1.6 Discrepancy between current behavior and goal<br>2.3 Self-monitoring of behavior<br>2.4 Self-monitoring of outcome(s) of behavior<br>2.7 Feedback on outcome(s) of behavior<br>3.1 Social support (unspecified)<br>4.1 Instruction on how to perform a behavior<br>7.1 Prompt/cues	1) Women in the intervention group had significantly lower GWG than women in the control group (10.21 kg vs 12.36 kg, *P* =<.001; Cohen's *d* =.39).<br>2) Women in the intervention group had a significantly lower weekly rate of GWG than women in the control group (mean between-group difference -0.07 kg per week, *P* <.001).<br>3) Proportion of women exceeding the IOM guidelines for weekly rate of GWG and total GWG was significantly lower in the intervention group than in the control group (48% vs 69%, *P* <.001 and 41% vs 66%, *P* <.001, respectively).<br>4) Proportion of women meeting the IOM guidelines for weekly rate of GWG and total GWG was significantly higher in the intervention group than in the control group (33% vs 24%, *P* =.059 and 36% vs 22%, *P* =.002, respectively).<br>5) Proportion of women gaining below the IOM guidelines for weekly rate of GWG and total GWG was significantly higher in the intervention group than in the usual care group (19% vs 8%, *P* <.001 and 23% vs 12%, *P* =.008, respectively).	Yes
P15		Adaptive, behavioral intervention impact on weight gain, physical activity, energy intake, and motivational determinants: results of a feasibility trial in pregnant women with overweight/obesity.	Symons Downs D, Savage JS, Rivera DE, et al.	2021	*Journal of Behavioral Medicine. 2021;44(5):605-621. doi:10.1007/s10865-021-00227-9*	Pilot RCT	Primary	1) To characterize participant compliance with the longitudinal data collection protocol and intervention implementation.<br>2) To describe frequency of exposure to the adaptive intervention dosages.<br>3) To determine pre-post change in GWG and explore secondary outcomes between the intervention and control groups.	Yes	- GA: 8 -12 weeks<br>- Age: 18 - 40 years<br>- BMI 24-45 kg/m2<br>- Singleton pregnancy<br>- English speaking<br>- Residing in / near Pennsylvania (US)<br>- Exclusion: diabetes at study entry, severe allergies or dietary restrictions, contraindications to prenatal PA, no pre-pregnancy overweight / obesity	Overweight & obese	31	Recruitment took place on-site in the clinic, and through community-based, and Web-based strategies (study Web site, Facebook ads)	USA	2016-2018	No	Participants were divided into an intervention and control group. All participants received CAU and followed the same measurement protocol, including daily, weekly, and monthly assessments. Participants uploaded daily weight data, monitored daily intake, and recorded food and beverage consumption. They wore a wrist-worn monitor daily and a waist-worn accelerometer in 2-week cycles to track PA throughout the study. Dietary intake was recorded via a mobile app on 2 weekdays and 1 weekend day. In addition, the intervention group received tailored components, including weekly meetings with a dietician, caloric goals, and booklets on healthy diet and PA. Every 3-4 weeks, the intervention dosage was reviewed and adjusted if needed, with a maximum of five dosage increases.	Theory of planned behavior	combined diet & PA	8 to 12	27	8 to 12	trim 1	Weekly onsite and/or remote live sessions (45-60 min/week), continuous monitoring (mHealth tools)	app, e-mail, pen-and-paper, face-to-face, mHealth tools	Digital-mixed	1,1 Goal setting (behavior)<br>1,2 Problem solving<br>1,3 Goal setting (outcome)<br>1,4 Action planning<br>1,7 Review outcome goals<br>2,2 Feedback on behavior<br>2,3 Self-monitoring of behavior<br>2,7 Feedback on outcomes of behavior<br>3,1 Social support (unspecified)<br>4,1 Instruction on how to perform a behavior<br>6,1 Demonstration of the behavior<br>10,1 Material incentive (behavior)	1) The intervention group gained 1.9 kg less than the control group, but this difference was not significant (*P* =.43).<br>2) In the intervention group PA from pre- to posttest increased, while in the control group decreased. However, this difference was not significant (*P* =.48).<br>3) Energy intake increased less from pre- to posttest for the intervention compared to the control group (*P* =.02).	NA

Primary data articles (continued)																													
ID	Group code	Title	Author(s)	Year	Publication details	Study type	Article type	Goal	IoM guidelines	Population details	Target group	Sample size	Port of entry	Country	Study year	Study during COVID-19	Description intervention	Theory	Lifestyle type	Enrollment GA	Intervention duration	Timing of intervention in GA weeks	Timing of intervention in trimester	Frequency	Delivery medium	Digital-only / Digital-mixed	BCTs used	Results	Success-full
P13		A Web-Based mHealth Intervention With Telephone Support to Increase Physical Activity Among Pregnant Patients With Overweight or Obesity: Feasibility Randomized Controlled Trial	Thomas T, Xu F, Sridhar S, et al.	2022	*JMIR Formative Research.* 2022;6(6):e33929.	Pilot RCT	Primary	1) To investigate whether it was feasible to implement the mHealth lifestyle intervention for promoting self-monitoring of weight and PA for women with overweight and obesity within the target population.<br>2) To to determine whether the intervention was acceptable to the study participants.<br>3) To explore preliminary efficacy findings (ie, PA and GWG) using adjusted intention-to-treat analyses.	Yes	- GA: <12 weeks<br>- Age: ≥ 21 years<br>- BMI: 25-40 kg/m2<br>- Singleton pregnancy<br>- Receiving care at Kaiser Permanente Northern California<br>- English speaking<br>- Exclusion: multiple gestation, pregnancy loss, high-risk pregnancy , thyroid disease, use of glucose-lowering medications or corticosteroids, no reliable access to smartphone and Wi-Fi at home.	Overweight & obese	68	Participants were recruited through the electronic health record	USA	2017-2018	No	Participants were divided into an intervention and control group. The control group received care as usual, including an early gestation visit (~7-10 weeks GA) and a newsletter on IOM GWG guidelines and advice on healthy eating. The intervention group were also asked to set PA goals, work up to 150 active minutes per week, wear a wrist-worn accelerometer, and weigh themselves daily. They received a baseline visit at home, monthly motivational check-in calls to review and reset goals, access to a Web site with data from the accelerometer and weighing scale, a GWG graph, and resources for managing GWG, and personal messages to motivate, remind and celebrate success.	Social cognitive theory; Trantheoretical model of change	combined diet & PA	< 12	29	10 to 12	trim 1	1 live session, monthly calls, weekly SMS / mail messages, on demand digital	SMS, e-mail, Web site, pen-and-paper, telephone, mHealth tools	Digital-mixed	1.1 Goal setting (behavior)<br>1.3 Goal setting (outcome)<br>1.5 Review behavioral goal(s)<br>1.6 Discrepancy between current behavior and goal<br>1.7 Review outcome goals<br>2.2 Feedback on behavior<br>2.3 Self-monitoring of behavior<br>2.4 Self-monitoring of outcome(s) of behavior<br>2.7 Feedback on outcome(s) of behavior<br>3.1 Social support (unspecified)<br>7.1 Prompts/cues<br>8.1 Behavioral practice / rehearsal<br>9.1 Credible source<br>10.4 Social reward	1) Participants in the intervention and the control group had the same total GWG (+1.14 kg, 95% CI -0.71 to 3.00, Cohen's *d* =.15).<br>2) Participants in the intervention and the control group had the same rate of GWG (+0.03 kg, 95% CI -0.02 to 0.09).	NA
S20		Weight-related SMS texts promoting appropriate pregnancy weight gain: a pilot study.	Pollak KI, Alexander SC, Bennett G, et al.	2014	*Patient Education and Counseling* . 2014;97(2):256-260. doi:https://doi.org/10.1016/j.pec.2014.07.030	Pilot RCT	Primary	To pilot an SMS-texting intervention to promote healthy GWG among overweight and obese women.	No	- GA: 12 - 21 weeks<br>- Age: ≥ 18 years<br>- BMI: 25 - 40 kg/m^2<br>- Exclusion: pre-existing diabetes, limited mobility or inability to walk, impaired cognition or mental health with inability to provide consent	Overweight & obese	33	Participants were recruited from two prenatal clinics	USA	2012	No	Participants were divided into an SMS-texting intervention (Preg CHAT) or a generic texting intervention (Txt4Baby). The Txt4Baby group received general pregnancy-related text message 3 days a week. The PregChat group received personalized feedback through text messages three days a week based on intake of sweetened beverages, fruits and vegetables, fast food, daily step count, and weight.	Social cognitive theory	combined diet & PA	12 to 21	16	16	trim 2	SMS on 3 days/ week for 16 weeks, 2 SMS / month	SMS	Digital-only	1.1 Goal setting (behavior)<br>2.2 Feedback on behavior<br>2.3 Self-monitoring of behavior<br>2.4 Self-monitoring outcomes of behavior<br>7.1 Prompts / cues<br>10.8 Incentive (outcome)	Participants in the intervention group gained 6 pounds less than those in the control group, but this was not statistically significant (*P* =.24).	NA
S15		Preventing excessive gestational weight gain among African American women: a randomized clinical trial	Herring SJ, Cruice JF, Bennett GG, Rose MZ, Davey A, Foster GD.	2016	*Obesity* . 2016;24(1):30-36. doi:https://doi.org/10.1002/oby.21240	RCT	Primary	To evaluate whether a technology-based, behavioral intervention could decrease the proportion of overweight or obese African American women who exceeded IOM guidelines for GWG.	Yes	- GA: < 20 weeks<br>- Age ≥ 18 years<br>- BMI: 25 - 45 kg/m^2<br>- Singleton pregnancy<br>- Self-identification as African American<br>- Medicaid recipient (income proxy)<br>- Exclusion: conditions requiring specialized nutritional care, endorsed current tobacco use	Overweight & obese	66	Recruitment took place at outpatient obstetric practices at Temple University	USA	2013-2014	No	Participants were divided in an intervention and control group. Both groups received CAU. The intervention group additionally received a technology-based behavioral intervention including guidance through personalized health coach calls, texts and feedback, a pedometer, and a walking DVD, on energy intake, PA, and weekly self-weighing. Also, in a Facebook group participants were encouraged to like weekly coach posts and provide updates.	Social ecological model; Social cognitive theory	combined diet & PA	< 20	28	8 to 17	trim 1 and 2	Daily SMS, 5-7 coaching calls, on-demand digital	SMS, social media, telephone	Digital-only	1.1 Goal setting (behavior)<br>1.3 Goal setting (outcome)<br>2.2 Feedback on behavior<br>2.3 Self-monitoring of behavior<br>2.4 Self-monitoring outcomes of behavior<br>2.7 Feedback on outcomes of behavior<br>3.2 Social support (practical)<br>3.3 Social support (emotional)<br>4.1 Instruction on how to perform a behavior<br>4.2 Information about antecedents<br>5.1 Information about health consequences<br>6.1 Deomstration of the behavior<br>6.2 Social comparison<br>7.1 Prompt/Cues<br>10.2 Material reward (behavior)<br>10.4 Social reward	1) Participants assigned to the intervention group were significantly less likely to exceed IOM guidelines compared to usual care (37% vs. 66%; *P* =.03; OR=.31).<br>2) Intervention participants gained less weight in pregnancy than controls (8.7 vs. 12.3 kg; *P* =.046; Cohen's *d* =.55).	Yes[a,b]
P100	O4	The effectiveness of an online intervention in preventing excessive gestational weight gain: the e-moms roc randomized controlled trial	Olson CM, Groth SW, Graham ML, Reschke JE, Strawderman MS, Fernandez ID.	2018	*BMC Pregnancy and Childbirth* . 2018;18(1). doi:https://doi.org/10.1186/s12884-018-1767-4	RCT	Primary	To evaluate effectiveness of a pregnancy intervention & combined pregnancy and postpartum intervention on weight retention at 12 months postpartum.	Yes	- GA : ≤ 20 weeks<br>- Age: 18-35 years<br>- Exclusion: BMI < 18.5 and ≥35 kg/m^2, multiple gestation, weight-affecting medical or psychiatric conditions, and no e-mail address<br><br>- Note that subjects were stratified in both income groups (low vs high) and BMI groups (normal vs overweight + obese)	Normal weight to moderately obese	1689	Pregnant women were screened by research staff in prenatal clinics, private obstetric practices, ultra-sound offices, and over the phone and online in a large Northeastern US city	USA	2011-2013	No	Participants were divided into 3 groups: placebo, pregnancy intervention & PP control, and pregnancy & PP intervention. All groups received standard care. Also, all groups received behavioral change tools on a study website & an app platform, but placebo group did not receive weight gain tracker, and diet & PA goal setting and self-monitoring tool.	Integrative model of behavior prediction; Behavior model for persuasive design	combined diet & PA	< 20	25	12 to 20	trim 1 and trim 2	Weekly reminders, on-demand digital, continuous monitoring (mHealth tools)	app, e-mail, Web site, mHealth tools	Digital-only	1.1 Goal setting (behavior)<br>1.2 Problem solving<br>1.3 Goal setting (outcome)<br>1.4 Action planning<br>2.4 Self-monitoring of outcomes of behavior<br>2.7 Feedback on outcome(s) of behavior<br>7.1 Prompt/cues<br>8.1 Behavioral practice / rehearsal<br>10.3 Non-specific reward	For a pregnancy intervention, the authors reported no significant difference in the proportion of women with excessive GWG in the intervention group versus the control group (48% vs 46%, RR 1.09, P=.12).	No
P7		A Promising Food-Coaching Intervention Program to Achieve Optimal Gestational Weight Gain in Overweight and Obese Pregnant Women: Pilot Randomized Controlled Trial of a Smartphone App	Li LJ, Aris IM, Han WM, Tan KH.	2019	*JMIR Formative Research* . 2019;3(4):e13013. doi:https://doi.org/10.2196/13013	Pilot RCT	Primary	To determine the feasibility of a novel food-coaching smartphone app for controlling gestational weight gain and macronutrient intake among overweight and obese pregnant women.	Yes	'- GA: 18 - 20 weeks<br>- Age: ≥ 21 years<br>- BMI: ≥ 25 kg/m^2<br>- Singapore-based<br>- Capable of reading and writing in English<br>- Exclusion: special dietary restrictions due to medical conditions	Overweight & obese	26	Recruitment took place through a subsidized clinic within a tertiary government hospital in Singapore	Singapore	2018-2019	No	Participants were divided into intervention and control groups. Both groups received standard dietary guidance for pregnancy at the time of recruitment. In addition, the intervention group received 8 weeks of real-time food coaching via a smartphone app.The intervention group could upload images of their meals, drinks, or desserts to the app and receive real-time, detailed feedback and guidance from professional dietitians throughout the day.	Not described	diet	18 to 20	8	18 to 20	trim 2	8 weekly live digital sessions, on-demand digital	app	Digital-only	2.3 Self-monitoring of behavior<br>2.2 Feedback on behavior<br>3.1 Social support (unspecified)<br>4.1 Instruction on how to perform a behavior<br>5.1 Information about health consequences<br>9.1 Credible source	1) More participants met the IOM guidelines in the intervention group than in the control group (4-week follow up: 58% vs 53%; 8-week follow-up: 67% vs 36%; not tested statistically).<br>2) Although not significant, women in the intervention group had less weight gain than those in the control group at both the 4-week and 8-week follow-up (-0.15 Kg, *P* =.83 and -0.08 kg, *P* =.92, respectively).	NA
P89		Remote gestational weight gain monitoring in a large low-risk US population	Litman EA, Kavathekar T, Amdur R, Sebastian A, Marko K.	2021	*Obesity science & practice* . 2021;8(2):147-152. doi:https://doi.org/10.1002/osp4.554	Real-world user data	Primary	To evaluate gestational weight gain tracking in a large low-risk obstetrical population using remote patient monitoring and a mobile phone app.	Yes	- GA: < 20 weeks<br>- Age: < 40 years<br>- Singleton pregnancy<br>- Lack of pre-chronic medical illness<br>- At least 3 weights recorded over more than 2 trimesters	Pregnant women with weight recordings	15.468	Enrollment happened directly via Babyscripts app	USA	2016-2020	No	There only was an intervention group and that used the BabyScripts app. The app is provided through healthcare professionals to track GWG. Participants could enter their weight manually or via a Bluetooth-connected scale. The app provides gives targeted, gestational-age-specific educational materials.	Not described	NA	< 20	29	< 20	trim 1 and trim 2	On-demand digital	app, mHealth tools	Digital-only	2.4 Self-monitoring of outcomes of behavior<br>5.1 Information about health consequences<br>9.1 Credible source (app provided by HCP)	1) Participants who were highly engaged with the mobile app had increased adherence to the IOM guidelines (29.8% vs. 9.4%, *P*< 0.001).<br>2) A larger proportion of highly engaged participants adhered to the IoM guidelines for rate of weight gain in trimesters 2 and 3, compared to the lowest engaged patients (12.7% vs. 6.8%, *P* < 0.001).	Yes[b,c]
P41	H2	Effectiveness of a Smartphone App to Promote Healthy Weight Gain, Diet, and Physical Activity During Pregnancy (HealthyMoms): Randomized Controlled Trial	Sandborg J, Söderström E, Henriksson P, et al.	2021	*JMIR mHealth and uHealth* . 2021;9(3):e26091. doi:https://doi.org/10.2196/26091	RCT	Primary	To investigate effectiveness of a 6-month intervention starting in GA week 14 on GWG in GA week 37.	Yes	- GA: ≤ 12 weeks<br>- Age: ≥ 18 years<br>- Singleton pregnancy<br>- Read and speak Swedish well enough to use the app.<br>- Exclusion: previous diagnosis of eating disorder, diabetes, or other medical conditions with possible effects on body weight	Healthy pregnant women	305	Participants were recruited in early pregnancy at the first routine visit at maternity clinics in the county of Östergötland, Sweden	Sweden	2017-2020	Yes	Participants were divided into intervention and control group. The control group received standard care (incl. optional lecture on healthy lifestyle) including repeated weight measurements. The intervention group received the same plus a 6-month app intervention program they could use as much as preferred. The program encouraged healthy diet and PA, for promoting healthy GWG, irrespective of pre-preg BMI. Push notifications were sent 4 times a week for information, support, strategies, guidance, encouraging info, and reminders.	Social cognitive theory	combined diet & PA	first routine maternity care visit	26	13 to 14	trim 1 and trim 2	4 push notications/ week, on-demand digital, 3 contact points (only for measurements)	app, mHealth tools	Digital-only	1.1 Goal setting (behavior)<br>1.3 Goal setting (outcome)<br>2.2 Feedback on behavior<br>2.3 Self-monitoring of behavior<br>2.4 Self-monitoring outcomes of behavior<br>2.5 Monitoring outcomes of behavior by others without feedback<br>2.7 Feedback on outcomes of behavior<br>3.2 Social support (practical)<br>3.3 Social support (emotional)<br>4.1 Instruction on how to perform a behavior<br>4.2 Information about antecedents<br>5.1 Information about health consequences<br>6.1 Demonstration of the behavior<br>7.1 Prompt/cues	1) Results showed no statistically significant effect on GWG between control and intervention (-0.2 kg, *P* =.62, Cohen's *d* =.28).<br>2) There was no statistical difference in adherence to the recommendations between intervention and control (50% vs 50%, *P* =.32).<br>3) However, results differed per BMI group: for women with overweight and obesity, GWG in the intervention group was lower than those in the control group (-1.67 kg, *P* =.03).<br>4) The intervention group had better diet quality than the control group (0.27; *P* =.017).	No
P42		Effectiveness of a Step Counter Smartband and Midwife Counseling Intervention on Gestational Weight Gain and Physical Activity in Pregnant Women With Obesity (Pas and Pes Study): Randomized Controlled Trial	Gonzalez-Plaza E, Bellart J, Arranz Á, Luján-Barroso L, Crespo Mirasol E, Seguranyes G.	2022	*JMIR Mhealth Uhealth.* 2022;10(2):e28886. doi: 10.2196/28886.	RCT	Primary	1) To evaluate the effectiveness of a complex digital health intervention, using a smartband and app with midwife counseling, on GWG and physical activity in women who are pregnant and have obesity and analyze its impact on maternal and perinatal outcomes.<br>2) To study the frequency of use, usability, and satisfaction with the mobile apps used by the women in the intervention group.	Yes	- GA: 12 - 18 weeks<br>- Age: ≥ 18 years<br>- BMI: ≥ 30 kg/m^2<br>- Singleton pregnancy<br>- Exclusion: women who had already used an app for monitoring physical activity and weight	Obese	120	Women who attended hospital obstetric clinics during prenatal care were recruited	Spain	2018-2020	Yes	Participants were divided into intervention and control groups. The intervention group used a smartband connected to an app to monitor physical activity and for communication with a midwife, who provided personalized health guidance. The control group received usual care (incl. health education on PA, GWG, and dietary habits.)	Social cognitive theory	combined diet & PA	12 to 18	21	12 to 28	trim 1 and trim 2	Bi-weekly app / SMS, monthly digital check-ins, continuous monitoring (mHealth tools), on-demand digital	app, SMS, mHealth tools	Digital-only	1.1 Goal setting (behavior)<br>1.3 Goal setting (outcome)<br>1.6 Discrepancy between current behavior and goal<br>2.3 Self-monitoring of behavior<br>2.4 Self-monitoring of outcome(s) of behavior<br>3.1 Social support (unspecified)<br>4.1 Instruction on how to perform a behavior<br>5.1 Information about health consequences<br>9.1 Credible source	1) Median GWG in the intervention group was significantly lower than in the control group (7.0 kg vs 9.3 kg, *P* =.04; Cohen's *d* =.42).<br>2) The adjusted mean GWG per week was significantly lower in the intervention group than in the control group (0.3 kg/week vs 0.5 kg/week, *P* =.008).	Yes
P108		The short-term effect of a mHealth intervention on gestational weight gain and health behaviors: The SmartMoms Canada pilot study	Souza SCS, Danilo, Nagpal TS, et al.	2022	*Physiology & Behavior* . 2022;257:113977-113977. doi:https://doi.org/10.1016/j.physbeh.2022.113977	Non-randomized intervention study	Primary	To assess the short-term effect of the SmartMoms Canada app to promote adequate GWG and healthy behaviors.	Yes	- GA: 12 - 20 weeks<br>- BMI: ≥18.5 and ≤39.9 kg/m2<br>- Singleton pregnancy<br>- No contraindications to exercise during pregnancy<br>- Able to communicate in English or French	Normal weight to moderately obese	27	Recruitment was carried out through online/internet sources, including social media platforms (i.e., Twitter, Instagram and Facebook sites).	Canada	unknown	Yes	As analyses focused on association between app use and GWG guidelines adherence, there was no control group. Participants were divided in higher/lower app usage groups.They got access to the SmartMoms Canada app, a Fitbit and a Withings scale. The app provided real-time feedback on nutrition, PA, sleep and GWG. Participants were encouraged to use it daily.	Transtheoretical model of change	combined diet & PA	12 to 20	9	12 to 20	trim 1 and trim 2	2 digital contact points, on-demand digital, continuous monitoring (mHealth tools)	app, mHealth tools	Digital-only	1.1 Goal setting (behavior)<br>1.2 Problem solving<br>1.5 Review behavior goal(s)<br>1.7 Review outcome goal(s)<br>2.2 feedback on behavior<br>2.3 Self-monitoring of behavior<br>2.4 Self-monitoring of outcome(s) of behavior<br>2.6 Biofeedback<br>2.7 feedback on outcome(s) of behavior<br>4.1 Instruction on how to perform a behavior<br>3.3 Social support (emotional)<br>7.1 Prompts/cues	1) Compared to participants in the lower app usage group, those in the higher app usage group better adhered to GWG guidelines, but this moderate effects was not statistically significant (Cramer's V=.21, P=.54).<br>2) Compared to participants in the lower app usage group, those in the higher app usage group had more moderate PA (mean difference: 8.41, *P* <.05) and MVPA (mean difference: 17.84, *P* <.05).	No
P40		Effectiveness of a nurse-led mHealth app to prevent excessive gestational weight gain among overweight and obese women: A randomized controlled trial	Chen HH, Lee CF, Huang JP, Hsiung Y, Chi LK.	2023	*Journal of Nursing Scholarship* . Published online September 19, 2022. doi:https://doi.org/10.1111/jnu.12813	RCT	Primary	To evaluate the effect of a mHealth-based program using an app + wearable activity tracker for managing & preventing eGWG among overweight & obese women.	Yes	- GA: < 17 weeks<br>- Age ≥ 20 years old<br>- BMI ≥ 25 kg/m^2<br>- Fluent in Mandarin / Chinese<br>- Exclusion: diagnosis of eating-related disorders, diabetes, and/or medical conditions that influence body weight	Overweight & obese	80	Participants were recruited from two prenatal clinics in northern Taiwan	Taiwan	2020	Yes	Participants were divided into intervention and control group. The intervention group used the MyHealthyWeight app and a wearable activity tracker, and the controls received standard antenatal treatments with no mHealth-based elements. The app features were designed for behavioral changes using theoretical-based techniques and included prenatal history, goal setting, chart history, records, prenatal information, rewards, reminders, and prenatal tools.	Social cognitive theory	combined diet & PA	< 17	19	< 17	trim 1 and trim 2	Weekly messages, on-demand digital, continuous monitoring (mHealth tools)	app, SMS, mHealth tools	Digital-only	1.3 Goal setting (outcome)<br>2.3 Self-monitoring of behavior<br>2.4 Self-monitoring of outcome(s) of behavior<br>2.7 Feedback on outcomes of behavior<br>7.1 Prompt/cues<br>10.1 Material incentive (behavior)<br>10.1 Reward (outcome)<br>10.4 Social reward	1) The rate of exceeding total GWG was not significantly different between control and intervention groups (22% vs 33%, *P* =.28).<br>2) However, in trimester 2 a significantly lower proportion of intervention participants exceeded their weekly GWG (45% vs 67%, *P* =.039). For trimester 1 and 3 they performed similar to the control group.<br>3) In trimester 3, obese women in the intervention group had less total GWG and body weight than those in the control group (-8.8 kg, *P* =.04; Cohen's *d* =.19 and -5.4 kg, *P* =.02, respectively). This did not hold for trimesters 1 and 2.	No
P38		Effect of the smartphone application on caesarean section in women with overweight and obesity: a randomized controlled trial in China	Feng Y, Shi C, Zhang C, Yin C, Zhou L.	2023	*BMC Pregnancy and Childbirth.* 2023;23(1). doi:https://doi.org/10.1186/s12884-023-06004-7	RCT	Primary	To evaluate the effect of the smartphone application, which aims to control the gestational weight gain, on the rate of CS in overweight and obese women.	Yes	'- Age: 20 - 40 years<br>- BMI: ≥ 24 kg/m^2<br>- Singleton pregnancy<br>- Primipara<br>- Exclusion: pre-pregnant diabetes, hypertension and/or other cardiovascular diseases, complications with systemic lupus erythematosus and other autoimmune diseases	Overweight & obese	268	Women were recruited at Beijing Obstetrics and Gynecology Hospital, Capital Medical University	China	2021-2022	Yes	Participants were divided into intervention (care as usual + app) and control group (care as usual). Intervention participants were asked to report their weight, diet and PA via the app at least once a week. The app contained sections for weight management (through weighing, diet and PA), daily recordings (with feedback), data trends, reminders, and pregnancy education per gestational week.	Not described	combined diet & PA	6 to 7	29	6 to 7	trim 1	Regular (daily/ weekly) reminders, on-demand digital, continuous monitoring (app)	app	Digital-only	2.3 Self-monitoring of behavior<br>2.4 Self-monitoring of outcome(s) of behavior<br>2.7 Feedback on outcome(s) of behavior<br>3.1 Social support (unspecified)<br>4.1 Instruction on how to perform a behavior<br>5.1 Information about health consequences<br>7.1 Prompts/cues<br>9.1 Credible source	Overall median GWG in the intervention group was significantly lower than that in the control group (8.5 kg vs 10.0 kg; *P* =.008, Cohen's *d* =.42).	Yes[a]

Primary data articles (continued)

ID	Group code	Title	Author(s)	Year	Publication details	Study type	Article type	Goal	IoM guidelines	Population details	Target group	Sample size	Port of entry	Country	Study year	Study during COVID-19	Description intervention	Theory	Lifestyle type	Enrollment GA	Intervention duration	Timing of intervention in GA weeks	Timing of intervention in trimester	Frequency	Delivery medium	Digital-only / Digital-mixed	BCTs used	Results	Success-full
P22		Characterising the use, users and effects of a health app supporting lifestyle changes in pregnant women	Koivuniemi E, Raats MM, Ollila H, Löyttyniemi E, Laitinen K.	2023	*British Journal Of Nutrition* . 2022;130(3):433-445. doi:https://doi.org/10.1017/s0007114522003439	Pilot RCT	Primary	1) To determine whether the addition of evidence-based information on health-promoting lifestyle delivered via the health app would exert an effect on the change in gestational weight, diet quality and physical activity during pregnancy.<br>2) To characterise the health app use and users among pregnant women.<br>3) To investigate whether the frequency of the health app use has an effect on the change in gestational weight, diet quality and physical activity.	No	- GA: < 28 weeks<br>- Fluent in Finnish	Pregnant women using mobile devices	1038	Women were recruited through social media announcements	Finland	2017-2018	No	Participants were divided into a 'standard' app group and 'enhanced' app group. They recorded lifestyle habits in the app and were able to monitor possible changes by viewing graphs of their recordings. Both groups received weekly reminders via the app that encouraged them to make recordings regularly. Women in the enhanced group received additional, non-personalised information on health-promoting lifestyle during pregnancy to inform and motviate them.	Social cognitive theory	combined diet & PA	< 28	26	9 to 20	trim 1 and trim 2	Weekly reminders, on demand digital, continuous monitoring (app)	app	Digital-only	1.1 Goal setting (behavior)<br>1.2 Problem solving<br>1.5 Review behavior goal(s)<br>1.7 Review outcome goal(s)<br>2.3 Self-monitoring of behavior<br>2.4 Self-monitoring of outcome(s) of behavior<br>4.1 Instruction on how to perform a behavior<br>4.2 Information about antecedents<br>5.1 Information about health consequences<br>7.1 Prompts / cues	1) Authors reported no significant differences in GWG or in the changes in IDQ scores or MET scores.<br>2) In the intervention group, the proportion of women with regular eating frequency (i.e. ≤2 meals skipped per week) was lower in late pregnancy as compared with early pregnancy (OR 0.47, *P* =.045), whereas in the control app group, there was no difference between early and late pregnancy in the proportion of women with a regular eating frequency (OR 1.44, *P* =.33).<br>3) The proportion of women with high and moderate activity decreased more in app non-users than in frequent app users (OR 0.61, *P* =0.025) and occasional app users (OR 0.55, *P* =0.04).	NA
P28		Development and feasibility of a web-based gestational weight gain intervention for women with pre-pregnancy overweight or obesity	Waring ME, Simas T a. M, Heersping GE, et al.	2023	*mHealth.* 2023;9:13.	Pilot RCT	Primary	To develop and examine the feasibility of a web-based gestational weight gain intervention.	Yes	- GA: <20 weeks<br>- Age: ≥ 18 years<br>- BMI: ≥ 25 & <45 kg/m2<br>- Singleton pregnancy<br>- Active Facebook users<br>- English speaking<br>- Plan to deliver at Umass Memorial Medical Center<br>- Medical clearance from provider<br>- Exclusion: chronical medical conditions or medication use influencing body weight, previous bariatric surgery, current participation in weight loss program	Overweight & obese	12	Participants were recruited from the obstetric practices at Umass Memorial Medical Center and the Worcester-ares community	USA	2016	No	As this was a feasibility study, there was no control group. Participants gained access to a Web site with a discussion board with peers and interventionists, and a weight gain tracker and online resources. Participants were encouraged to check the feed daily, to regularly post questions, successes and challenges, and to reply to other's posts. They were also encouraged to track their diet and activity, and to weigh themselves at least weekly. The interventionist posted messages daily and checked the website twice a day for interacting asynchronously with participants, and to provide support, help problem-solve challenges, and encourage engagement.	Social cognitive theory	combined diet & PA	< 20	12	14 to 18	trim 2	On demand dgitital, regular reminders	Web site	Digital-only	1.1 Goal setting (behavior)<br>1.2 Problem solving<br>1.3 Goal setting (outcome)<br>1.6 Descrepancy between current behavior and goal<br>2.3 Self-monitoring of behavior<br>2.4 Self-monitoring of outcome(s) of behavior<br>3.2 Social support (practical)<br>3.3 Social support (emotional)<br>4.1 Instruction on how to perform a behavior<br>6.1 Demonstration of the behavior<br>7.1 Prompts/cues<br>9.1 Credible source<br>12.1 Restructuring the physical environment<br>12.3 Restructuring the social environment	The authors reported that 70% of participants had eGWG, 10% had inadequate GWG, and 20% gained within the recommended ranges.	NA
P52		Feasibility of Telehealth and Innovative Technologies to Limit Excessive Gestational Weight Gain	Mattson R, Barger MK.	2024	Nursing for Women S Health. 2023;28(1):30-40.	Pilot RCT	Primary	To test the feasibility of using telehealth to deliver nutritional counseling by tracking gestational weight gain remotely using Bluetooth weight scales in pregnant women with obesity.	Yes	- GA: >12 weeks & <27 weeks<br>- Age: ≥ 18 years<br>- BMI: ≥ 30kg/m2<br>- Singleton pregnancy<br>- English speaking<br>- Exclusion: no acces to internet, (history of gestational) diabetes, history of gestational hypertension or preeclampsia	Obese	22	Participants were recruited from 2 healthcare centers in a large metropolitan area	USA	2021	Yes	Participants were divided in two intervention groups: a self-weighing group (WA) and a self-weighing + counseling group (WC). In addition, there was an historical control group. All intervention participants were asked to weigh themselves at least once a week. The WC group also received 30 min of online counseling once a week for 6 weeks. Sessions consisted of tailored counseling on healthy diet using motivational interviewing, and included review of previous week's diet, identification of needed nutrients, sample meal plans, education on healthy food choices, and strategies for healthy eating.	Not described	diet	12 to < 27	6	10 to 25	trim 1 and 2	6 online sessions (30 min)	Telehealth system, mHealth tools	Digital-only	1.1 Goal setting (behavior)<br>1.3 Goal setting (outcome)<br>2.2 Feedback on behavior<br>2.3 Self-monitoring of behavior<br>3.1 Social support (unspecified)<br>3.2 Social support (practical)<br>4.1 Instruction on how to perform a behavior<br>5.1 Information about health consequences<br>9.1 Credible source	1) Participants in the combined intervention groups gained less weight than those in the control group, but this effect was not significant (-0.7 lb, *P* =.72).<br>2) Participants in the WC group gained less than those in the WA alone group, but this effect was not significant (-1.5 lb, *P* =.52).<br>3) Participants in the combined interventions groups who weighed themselves ≥6 times / week gained less weight than those who weighed <6 times / week, but this effect was not significant (-2.7 lb, *P* =.99)	NA

Secondary data articles

ID	Group code	Title	Author(s)	Year	Publication details	Study type	Article type	Goal	IoM guidelines	Study selection / population details	Target group	#Articles included	(Average) Sample size	Overlap primary articles	Time period	Study during COVID-19	Description intervention	Theory	Lifestyle type	Enrollment GA	Intervention duration	Timing of intervention in GA weeks	Timing of intervention in trimester	Frequency	Delivery medium	Digital-only / Digital-mixed	BCTs used	Results	Success-full
E68		Technology-supported dietary and lifestyle interventions in healthy pregnant women: A systematic review	O'Brien OA, McCarthy M, Gibney ER, McAuliffe FM.	2014	*European Journal of Clinical Nutrition* . 2014;68(7):760-766. doi:https://doi.org/10.1038/ejcn.2014.59	Systematic review	Secondary	To systematically review the literature on the use of technology-supported lifestyle interventions for healthy pregnant women and their impact on maternal outcomes.	NA	Technology-supported lifestyle interventions for healthy GWG (because of lack of published RCTs, cross-sectional observational studies, feasibility studies and ongoing trials were included)	All healthy pregnant women (without pregnancy-related conditions)	7	2833	0	2008-2013	NA	NA	NA	NA	NA	NA	NA	NA	NA	NA	NA	NA	1) Technology-supported lifestyle interventions in pregnancy hold potential as a safe and sustainable adjunct to traditional health-care models.<br>2) The quality and quantity of published evidence to support the use of such interventions is low.<br>3) Findings raise the issue of uptake levels and socio-cultural acceptance of such lifestyle interventions.	NA
P34	O2	Does Usage of an eHealth Intervention Reduce the Risk of Excessive Gestational Weight Gain? Secondary Analysis From a Randomized Controlled Trial	Graham ML, Strawderman MS, Demment M, Olson CM.	2017	*Journal of Medical Internet Research* . 2017;19(1):e6. doi:https://doi.org/10.2196/jmir.6644	Secondary analysis of RCT data	Secondary	To create a novel measure of Internet-based intervention usage patterns and examine whether usage of an Internet-based intervention is associated with reduced risk of excessive GWG.	NA	See P100	See P100	NA	1335	NA	NA	NA	NA	NA	NA	NA	NA	NA	NA	NA	NA	NA	NA	1) In control group there were 3 different types of patterns of app usage by the participants. In intervention group there were 5.<br>2) In control arm, GWG outcomes did not differ by usage pattern. In interventions group GWG outcomes did differ per usage pattern.<br>2a) In lower income-normal BMI group, 'almost consistent or inconsistent' trackers (~3/4 tracked data, but not goals) had risk of eGWG and 'inconsistent' trackers gained more than 'nonuser' (didn't track) usage pattern.<br>2b) In higher income-normal BMI group 'consistent' trackers (tracked data, read some info, didn't set goals) had lower risk of eGWG rate than 'nonusers'.<br>2c) In higher income-high BMI group 'consistent' trackers gained less than 'nonusers'.<br>3) Compared to participants with lower usage patterns, participants in higher usage patterns and higher income gained less, both in normal and high BMI subgroups (total mean GWG -1.83 kg). For participants with lower income this difference did not exist.	Yes
S4		Effect of diet and physical activity based interventions in pregnancy on gestational weight gain and pregnancy outcomes: meta-analysis of individual participant data from randomised trials.	International Weight Management in Pregnancy (i-WIP) Collaborative Group.	2017	*BMJ.* 2017;358:j3119	Meta-analysis	Secondary	To synthesise the evidence on the overall and differential effects of interventions based on diet and physical activity during pregnancy.	NA	R(C)Ts that assessed the effects of interventions based on diet, physical activity, and mixed interventions in pregnancy, on maternal and offspring outcomes.	All pregnant women except those with GDM	81	215	2	1992-2017	NA	NA	NA	NA	NA	NA	NA	NA	NA	NA	NA	NA	1) Based on IPD data, diet and physical activity based interventions resulted in significantly less gestational weight gain compared with control (mean GWG -0.70 kg).<br>2) When supplementing IPD data with study level data from non-IPD studies, the difference between intervention and control increased (mean GWG -1.1 kg), but so did heterogeneity.<br>3) No evidence was found of differential intervention effects across subgroups.	Yes
S19		Electronic-based lifestyle interventions in overweight or obese perinatal women: a systematic review and meta-analysis	Lau Y, Klainin-Yobas P, Htun TP, et al.	2017	*Obesity Reviews* . 2017;18(9):1071-1087. doi:https://doi.org/10.1111/obr.12557	Meta-analysis	Secondary	To synthesize the best evidence to assess the effectiveness of e-based lifestyle interventions in improving maternal and neonatal outcomes among perinatal overweight or obese women.	NA	Any types of RCTs on e-based lifestyle interventions	Overweight & obese	17	226	2	2006-2016	NA	NA	NA	NA	NA	NA	NA	NA	NA	NA	NA	NA	1) Participants in the intervention group had lower GWG than controls (GWG -0.63 kg, *P* =.004).<br>2) The e-based lifestyle interventions incorporating in-person, phone or a combination of in-person and phone delivery formats were found to be effective for reducing GWG (*P* =.004 for all 3) while no such effect was found for solely ebased platforms (*P* =.27). No significant effect was found for subgroup differences.	Yes
S17	O3	Association between consistent weight gain tracking and gestational weight gain: Secondary analysis of a randomized trial.	Olson CM, Strawderman MS, Graham ML.	2017	*Obesity* . 2017;25(7):1217-1227. doi:https://doi.org/10.1002/oby.21873	Secondary analysis of RCT data	Secondary	To examine the relationship between electronic weight gain tracking and weight gain outcomes in a sample of pregnant women in the United States.	NA	See P100	See P100	NA	898	NA	NA	NA	NA	NA	NA	NA	NA	NA	NA	NA	NA	NA	NA	1) 16.5% of low-income women and 34.2% of not-low-income women consistently tracked GWG.<br>2) More highly educated, older, and white women were more likely to be consistent GWG trackers.<br>3) Among not-low-income women, consistent GWG tracking was associated with 2.35 kg less GWG (*P* <.001) and reduced risk of eGWG (RR 0.73, *P*= .002).	Yes
S14		The effectiveness of eHealth technologies on weight management in pregnant and postpartum women: systematic review and meta-analysis	Sherifali D, Nerenberg KA, Wilson S, et al.	2017	*Journal of Medical Internet Research* . 2017;19(10). doi:https://doi.org/10.2196/jmir.8006	Meta-analysis	Secondary	To assess the effectiveness of eHealth technologies for weight management during pregnancy and the postpartum period and to review the efficacy of eHealth technologies on health behaviors, specifically nutrition and physical activity.	NA	eHealth weight management interventions with a specif ic goal of targeting GWG during pregnancy	All pregnant women	10	53	4	2012-2016	NA	NA	NA	NA	NA	NA	NA	NA	NA	NA	NA	NA	Meta-analysis on 6 studies showed a nonsignificant reduction in GWG (mean GWG -1.62 kg, *P* =.10) after exposure to the intervention.	No
P110		The Usability and Effectiveness of Mobile Health Technology-Based Lifestyle and Medical Intervention Apps Supporting Health Care During Pregnancy: Systematic Review	Overdijkink SB, Velu AV, Rosman AN, van Beukering MD, Kok M, Steegers-Theunissen RP.	2018	*JMIR mHealth and uHealth* . 2018;6(4):e109. doi:https://doi.org/10.2196/mhealth.8834	Systematic review	Secondary	To evaluate the usability, that is, feasibility and acceptability, as well as effectiveness of mHealth lifestyle and medical apps to support health care during pregnancy in high-income countries.	NA	RCTs, pilot studies, pro/retrospective cohort studies, surveys, and qualitative health care researchs on mHealth technology–based apps for pregnant women with the aim to support lifestyle and health care during pregnancy.	All pregnant women	29	1272	3	2008-2016	NA	NA	NA	NA	NA	NA	NA	NA	NA	NA	NA	NA	1) mHealth lifestyle apps and mHealth medical apps seem feasible and acceptable.<br>2) Evidence of effectiveness is limited because of small sample sizes.<br>3) Formal guidelines for quality certification of apps need to be developed.	NA
S22		Attenuating pregnancy weight gain—what works and why: a systematic review and meta-analysis.	Walker R, Bennett C, Blumfield M, et al.	2018	*Nutrients* . 2018;10(7):944. doi:https://doi.org/10.3390/nu10070944	Meta-analysis	Secondary	To address gaps in the literature by comparing the efficacy of all approaches that have been used to prevent excessive GWG and to further describe and explore characteristics that can be analyzed across intervention types.	NA	RCTs of all interventions designed to prevent excessive GWG.	All pregnant women except those with diabetes / GDM	89	285	4	2000-2017	NA	NA	NA	NA	NA	NA	NA	NA	NA	NA	NA	NA	1) Women in dietary interventions gained significantly less weight than those in the control group (mean GWG -3.27 kg, *P* <.001).<br>2) Women in PA interventions gained significantly less weight than those in the control group (mean GWG -1.02 kg, *P* <.001).<br>3) Women in lifestyle interventions (diet and PA combined) gained significantly less weight than those in the control group (mean GWG -0.73 kg, *P* <.001).<br>4) Women in eHealth interventions gained significantly less weight than those in the control group (mean GWG -2.26 kg, *P* <.001).<br>5) Interventions delivered to groups of women and those with a group component were more often effective (effective 62.5%, ineffective 37.35%; *P* =0.02) than interventions delivered to women individually (effective 33.3%, ineffective 66.7%; *P* =0.04).<br>6) The study did not find an optimal duration, frequency, intensity, delivery method or diet for preventing eGWG.	Yes

W63		Effects of Social Media and Mobile Health Apps on Pregnancy Care: Meta-Analysis	Chan KL, Chen M.	2019	*JMIR mHealth and uHealth.* 2019;7(1):e11836. doi:https://doi.org/10.2196/11836	Meta-analysis	Secondary	To examine the effectiveness of mHealth apps and social media interventions for pregnant and postpartum women.	NA	(Quasi)-experimental interventions providing pregnancy care using advanced technology and aimed at health outcomes	All pregnant women	16	225	3	2014-2018	NA	NA	NA	NA	NA	NA	NA	NA	NA	NA	NA	NA	There was a moderate effect in maternal weight control and maintaining optimal body composition by promoting lifestyle change and self-monitoring via mHealth apps and social media (d=0.45)	Yes
P36		Effect of Lifestyle Coaching Including Telemonitoring and Telecoaching on Gestational Weight Gain and Postnatal Weight Loss: A Systematic Review	Mertens L, Braeken MAKA, Bogaerts A.	2019	*Telemedicine and e-Health* . 2019;25(10):889-901. doi:https://doi.org/10.1089/tmj.2018.0139	Systematic review	Secondary	To explore the existing literature about the effect of technology-supported lifestyle interventions including telemonitoring and–coaching on GWG and PPWR.	NA	Technology-supported lifestyle interventions (pilot)-RCTs) including at least a mobile or a Web-based application aiming at improving at least GWG or PPWR and at least one of the following: PA, healthy eating, and/or psychological well-being	All healthy pregnant women	11	289	2	2012-2017	NA	NA	NA	NA	NA	NA	NA	NA	NA	NA	NA	NA	1) Technology-supported lifestyle interventions might affect GWG and PPWR, but more research is needed to examine the effectiveness, the usability, and the critical features of these interventions.<br>2) Interventions positively influence GWG and PPWL, but results are not always significant. Moreover, effects on PA and healthy eating are inconsistent.	NA

Secondary data articles (continued)

ID	Group code	Title	Author(s)	Year	Publication details	Study type	Article type	Goal	IoM guidelines	Study selection / population details	Target group	#Articles included	(Average) Sample size	Overlap primary articles	Time period	Study during COVID-19	Description intervention	Theory	Lifestyle type	Enrollment GA	Intervention duration	Timing of intervention in GA weeks	Timing of intervention in trimester	Frequency	Delivery medium	Digital-only / Digital-mixed	BCTs used	Results	Success-full
S27	O1	Use of an online diet goal-setting tool: relationships with gestational weight gain	Olson CM, Strawderman MS, Graham ML.	2019	*Journal of Nutrition Education and Behavior*. 2019;51(4):391-399.	Secondary analysis of RCT data	Secondary	1) To describe the use by pregnant women of an online dietary behavior change tool that used a 4-step goal-setting process and was aimed at preventing excessive GWG.<br>2) To determine the association between use of the diet goal-setting tool and excessive GWG, controlling for the characteristics of women that predicted use of the tool.	Yes	See P100	See P100	NA	898	NA	NA	NA	NA	NA	NA	NA	NA	NA	NA	NA	NA	NA	NA	1) Among women with normal BMI, setting ≥2 goals and engaging in self-monitoring were significantly (*P*= .03) associated with less GWG. Also, for this group the risk for excessive GWG was significantly reduced (*P* =.04).<br>2) Among women with higher BMI, setting ≥2 goals was significantly associated with greater GWG (*P* =.01), and wowen in this group were at significantly increased risk for excessive GWG (*P* =.03).	Yes
S31		Interventions including a nutrition component aimed at managing gestational weight gain or postpartum weight retention: a systematic review and meta-analysis.	Vincze L, Rollo M, Hutchesson M, et al.	2019	*JBI Database of Systematic Reviews and Implementation Reports* . 2019;17(3):297-364. doi:https://doi.org/10.11124/jbisrir-2017-003593	Meta-analysis	Secondary	To evaluate the effectiveness of interventions that include a nutrition component aimed at improving gestational weight gain and/or postpartum weight retention.	NA	Any kind of RCTs with a dietary component aimed at managing GWG	(Gestational) diabetes	48	206	2	1998-2016	NA	NA	NA	NA	NA	NA	NA	NA	NA	NA	NA	NA	1) A total of 12 out of 25 studies (48%) reported significant reductions in GWG.<br>2) Despite heterogeneity, pregnant women in the intervention groups gained less weight than those in the control groups (mean GWG -1.25 kg, *P* =.004).	Yes
P70		Mobile Phone-Based Behavioral Interventions in Pregnancy to Promote Maternal and Fetal Health in High-Income Countries: Systematic Review	Hussain T, Smith P, Yee LM.	2020	*JMIR mHealth and uHealth* . 2020;8(5):e15111. doi:https://doi.org/10.2196/15111	Systematic review	Secondary	To critically evaluate the existing literature about lifestyle interventions including telemonitoring with self-monitoring and telecoaching in mobile and Web-based applications for pregnant and postpartum women, aiming at achieving GWG in the range of the IOM guidelines for weight gain in pregnancy and at limiting PPWR.	NA	Stand-alone mobile phone–based interventions including, but not limited to, mobile phone apps, text messaging, games, and information services (meta-analyses, systematic reviews, RCTs and observational studies).	All pregnant women who used pregnancy-related mobile phone interventions	28	91	5	2014-2018	NA	NA	NA	NA	NA	NA	NA	NA	NA	NA	NA	NA	1) In high-income countries, utilization of mobile phone–based health behavior interventions in pregnancy demonstrates some correlation with positive beliefs, behaviors, and health outcomes.<br>2) More effective interventions are multimodal in terms of features and tend to focus on healthy gestational weight gain.	NA
S5		Supporting women of childbearing age in the prevention and treatment of overweight and obesity: a scoping review of randomized control trials of behavioral interventions.	Hutchesson MJ, de Jonge Mulock Houwer M, Brown HM, et al.	2020	*BMC women's health* . 2020;20(1):14. doi:https://doi.org/10.1186/s12905-020-0882-3	Scoping review	Secondary	To examine the extent and range of research undertaken to evaluate behavioral interventions to support women of childbearing age to prevent and treat overweight and obesity.	NA	Interventions promoting behavior change to prevent weight gain or overweight and obesity, or achieve weight loss or weight loss maintenance	All pregnant women	90	302	4	1998-2018	NA	NA	NA	NA	NA	NA	NA	NA	NA	NA	NA	NA	1) The majority of research on behavioral interventions that support women of childbearing age has focused on weight management during or after a pregnancy event.<br>2) There is a research gap to support weight management in the young adult female population in preconception and unrelated to pregnancy to improve their own chronic disease health trajectories.<br>3) Future research to examine delivery modes and mediums, optimal intervention duration and intensity, involvement of health care providers, and involvement of underrepresented populations should be considered for effectiveness and scalability.	NA
P51		Exclusively Digital Health Interventions Targeting Diet, Physical Activity, and Weight Gain in Pregnant Women: Systematic Review and Meta-Analysis	Rhodes A, Smith AD, Chadwick P, Croker H, Llewellyn CH.	2020	*JMIR Mhealth Uhealth.* 2020;8(7):e18255. doi: 10.2196/18255.	Meta-analysis	Secondary	1) To determine the effectiveness of exclusively digital diet and physical activity interventions to improve lifestyle behaviors or avoid eGWG.<br>2) To investigate user engagement with the interventions.<br>3) To identify and assess the usage of BCTs within the interventions.	NA	Digital interventions targeting dietary behaviors or physical activity in pregnancy, with the aim of improving diet or physical activity during pregnancy or managing GWG	All pregnant women, except for those with physical or mental health issues that would preclude them from participating in a diet- or physical activity-based	11	298	5	2012-2019	NA	NA	NA	NA	NA	NA	NA	NA	NA	NA	NA	NA	1) The meta-analyses showed no significant benefit of interventions on total GWG for either intention-to-treat (-0.28 kg, *P* =.63) data or per-protocol data (-0.65 kg, *P* =.34).<br>2) 7 BCTs were common to all effective interventions.<br>3) Effective interventions averaged over twice as many BCTs from the goals and planning, and feedback and monitoring domains as ineffective interventions.<br>4) There was a positive association between high engagement with key BCTs and greater intervention effectiveness.<br>5) Interventions using proactive messaging and feedback appeared to have higher levels of engagement.	No
E83		Mobile apps for self-management in pregnancy: a systematic review	Iyawa GE, Dansharif AR, Khan A.	2021	*Health and Technology* . 2021;11(2):283-294. doi:https://doi.org/10.1007/s12553-021-00523-z	Systematic review	Secondary	1) To systematically review and appraise the literature on mobile apps for self-management during pregnancy to identify the different self-management mobile apps which have been developed and evaluated to support women during pregnancy.<br>2) To identify the functionalities of self -management mobile apps for pregnancy.<br>3) To describe the effectiveness of self-management mobile apps for pregnancy.<br>4) To identify the research gaps in the current literature and suggesting directions for future research.	NA	Studies on feasibility, pilot testing or evaluation of apps for improving health outcomes in pregnant women	All pregnant women	18	127	1	2016-2019	NA	NA	NA	NA	NA	NA	NA	NA	NA	NA	NA	NA	1) The use of mobile apps during pregnancy point towards a positive impact on pregnancy and health service delivery.<br>2) Mobile apps can facilitate communication between pregnant women and medical practitioners despite the distance which is a suitable option for patients in areas with less access to medical pracitioners and medical facilities.	NA
S26		Effect of technology-supported interventions on prenatal gestational weight gain, physical activity, and healthy eating behaviors: a systematic review and meta-analysis.	Leonard, K. S., Evans, M. B., Oravecz, Z., Smyth, J. M., & Symons Downs, D.	2021	*Journal of Technology in Behavioral Science.* 2021;6:25-41.	Meta-analysis	Secondary	1) To examine the general effects of technology-supported interventions on prenatal GWG, physical activity, and healthy eating behaviors.<br>2) To descriptively examine intervention characteristics that may influence the effect of technology-supported interventions on regulating prenatal GWG and/or increasing physical activity/healthy eating behaviors.	NA	RCTs examining the influence of a technology-supported interventions on GWG, physical activity, and/or healthy eating behaviors	All pregnant women	21	298	6	2006-2019	NA	NA	NA	NA	NA	NA	NA	NA	NA	NA	NA	NA	1) Women in the technology-supported intervention had significantly lower mean GWG than controls (mean GWG -1.18, d=0.23).<br>2) The relatively small effects may be improved by intervention characteristics such as delivery mode (e.g., technology plus face-to-face), type of technology (e.g., tracking tools), and frequency of technology prescribed (e.g., daily).	Yes
S11		Efficacious lifestyle interventions for appropriate gestational weight gain in women with overweight or obesity set in the health care system: a scoping review.	Barroso CS, Yockey A, Degon E, et al.	2022	*The Journal of Maternal-Fetal & Neonatal Medicine.* 2022;35(25): 6411-6424.	Scoping review	Secondary	To systematically identify randomized controlled trials for appropriate GWG in women with overweight or obesity that were set in a health care system.	NA	RCTs of efficacious lifestyle interventions for appropriate GWG in pregnant women that were set in the health care system and delivered by non-clinicians	Overweight & obese	8	523	1	2011-2019	NA	NA	NA	NA	NA	NA	NA	NA	NA	NA	NA	NA	1) 4 of 8 identified trials had lifestyle interventions that were effective in improving GWG.<br>2) Effective interventions were all intensive in terms of frequency, included in-person sessions, were initiated early-to-mid pregnancy, and lasted (almost) the remaining pregnancy duration.<br>3) Lifestyle coaches trained in behavior change and motivational interviewing can facilitate in-person sessions, helping women set small goals and use self-monitoring strategies, and then provide them with feedback.	NA
P116	H1	User engagement in relation to effectiveness of a digital lifestyle intervention (the HealthyMoms app) in pregnancy	Henriksson P, Migueles JH, Söderström E, Sandborg J, Maddison R, Löf M.	2022	*Scientific Reports.* 2022;12(1). doi:https://doi.org/10.1038/s41598-022-17554-9	Secondary analysis of RCT data	Secondary	1) To determine the effectiveness of a 6-month mHealth intervention (the HealthyMoms app) on gestational weight gain, diet quality and physical activity.<br>2) To examine the associations of user engagement with the HealthyMoms app with gestational weight gain, diet quality and physical activity in pregnancy.	NA	See P41	See P41	NA	134	NA	NA	NA	NA	NA	NA	NA	NA	NA	NA	NA	NA	NA	NA	1) A greater number of registrations within the HealthyMoms app was associated with lower gestational weight gain and improved diet quality. These results were mainly attributable to the number of physical activity registrations (the more registrations, the lower the weight gain and the better the diet quality).<br>2) The number of app sessions and page views were not associated with gestational weight, diet quality and physical activity.	Yes
S29		Prevention of gestational diabetes mellitus and gestational weight gain restriction in overweight/obese pregnant women: a systematic review and network meta-analysis.	Wu S, Jin J, Hu KL, Wu Y, Zhang D.	2022	*Nutrients.* 2022;14(12):2383. doi:https://doi.org/10.3390/nu14122383	Meta-analysis	Secondary	To evaluate the effectiveness and identify the optimal intervention strategy to prevent GDM and restrict GWG among overweight/obese pregnant women.	NA	RCTs that evaluated lifestyle and medication interventions measuring GWG	Overweight & obese	23	386	2	2008-2021	NA	NA	NA	NA	NA	NA	NA	NA	NA	NA	NA	NA	Compared with usual care, women with PA, diet, and combined diet + PA interventions all gained less weight during pregnancy (mean GWG -1.98 kg, -1.95, and -1.21 kg, respectively).	Yes
P18		App-Supported Lifestyle Interventions in Pregnancy to Manage Gestational Weight Gain and Prevent Gestational Diabetes: Scoping Review	Raab R, Geyer K, Zagar S, Hauner H.	2023	*Journal of Medical Internet Research* . 2023;25:e48853. doi:https://doi.org/10.2196/48853	Scoping review	Secondary	1) To assess the scope of the available literature in the field.<br>2) To determine the concepts and characteristics of studies and their findings on GWG and GDM outcomes.<br>3) To describe the functionalities of available eHealth and mHealth apps.<br>4) To identify knowledge gaps, inform ongoing and future intervention projects, and provide a basis for systematic reviews and meta-analyses.	NA	(Quasi-)RCTs of app-supported lifestyle interventions in pregnancy and related qualitative and quantitative research across the different study phases	All pregnant women	97	Catergorized as:<br>≤ 100<br>> 100 - 1000<br>> 1000	13	2011-2022	NA	NA	NA	NA	NA	NA	NA	NA	NA	NA	NA	NA	1) In 7 of 18 included (pilot) RCTs, the rates of excessive GWG or total GWG could be reduced by the intervention.<br>2) The effectiveness and implementability of app-supported interventions have yet to be determined.<br>3) Identifying the most beneficial app features and intervention components is challenging. Consistent and comprehensive intervention and outcome reporting is needed.	NA

[a]Lower total average or median GWG; [b]Greater adherence to IOM GWG guidelines; [c]Lower weekly rate of GWG; [d]Lower risk of gaining excessive weight. Abbreviations: (e)GWG: (excessive) Gestational Weight Gain; GA: Gestational Age; PA: Physical Activity; trim: trimester; MVPA: Moderate-Vigorous Physical Activity; RCT: Randomized Controlled Trial; USA: United States of America; UK: United Kingdom; IOM: Institute of Medicine; CAU: Care As Usual; IDQ: Index of Diet Quality; MET: Metabolic Equivalent of Task; OR: Odds Ratio; HPD: Hypertensive Disorders of Pregnancy; SCT: Social CognitiveTheory; PP:Postpartum; RR: Relative Risk; DASH: Dietary Approach to Stop Hypertension; HbA1c: Hemoglobine A1c; LS mean: Least Square mean; GDM: Gestationa; Diabetes Mellitus; IPD: Individual Participant Data; BCT: Behavioral Change Technique; PPWR: Post-Partum Weight Retention; PPWL: Post-Partum Weight Loss
